# Photoluminescent-assisted hierarchical metafabric sunshade for high-efficiency passive cooling

**DOI:** 10.1016/j.xinn.2026.101503

**Published:** 2026-07-09

**Authors:** Shiliang Zhang, Mingxiang Liu, Jingwen Wang, Mingyue Chen, Jiawei Wu, Jun Liang, Zitan Yang, Wei Wei, Yaoguang Ma, Cheng-Wei Qiu, Huamin Zhou, Guangming Tao

**Affiliations:** 1 Research Center for Intelligent Fiber Devices and Equipment, School of Materials Science and Engineering, Huazhong University of Science and Technology, Wuhan 430074, China; 2 Wuhan National Laboratory for Optoelectronics, Huazhong University of Science and Technology, Wuhan 430074, China; 3 ReguLambda (Wuhan), Inc., Wuhan 430070, China; 4 School of Optics and Electronic Information, Huazhong University of Science and Technology, Wuhan 430074, China; 5 SAIC GM Wuling Automobile Co., Ltd., Liuzhou 545003, China; 6 State Key Laboratory for Extreme Photonics and Instrumentation, College of Optical Science and Engineering, Intelligent Optics and Photonics Research Center, Jiaxing Research Institute, ZJU-Hangzhou Global Scientific and Technological Innovation Center, International Research Center for Advanced Photonics, Zhejiang University, Hangzhou 310058, China; 7 Department of Electrical and Computer Engineering, National University of Singapore, Singapore 117583, Singapore; 8 Center for Intelligent Health Interdisciplinary Science, Central China Normal University, Wuhan 430079, China

**Keywords:** passive cooling, hierarchical structure, metafabric

## Abstract

High-rise buildings and electric vehicles in high-solar-irradiation regions consume large amounts of energy for climate control, posing a significant challenge to global sustainability. The primary obstacle lies in integrating zero-energy, efficient cooling solutions without compromising comfort. As urbanization intensifies, innovative solutions must balance environmental impact with functionality. We propose a holistic strategy featuring a photoluminescent-assisted hierarchical metafabric sunshade for post-glass spectral regulation. This material leverages to achieve a 95.8% emissivity in the mid-infrared (MIR) band and 94.3% reflectivity in the solar band. These particles efficiently convert absorbed ultraviolet (UV) light into visible light with an absolute quantum yield of 16.4%, significantly boosting cooling performance beyond traditional passive radiative materials. In real-world testing, the metafabric sunshade achieved a temperature reduction of 17.8°C and 14.4°C compared to the vehicles with blank and commercial sunshades, respectively. These results suggest that photoluminescent-enhanced metafabrics represent a sustainable, energy-saving pathway for cooling high-rise infrastructure and transportation, offering a scalable solution to mitigate urban heat islands and advance global sustainability goals.

## Public summary

• A novel strategy is proposed for post-glass spectral regulation inside high-rise buildings and vehicles.
• The metafabric sunshade achieves a high solar reflectivity (94.3%) and mid-infrared (MIR) emissivity (95.8%).
• The vehicle with the metafabric sunshade interior temperature exhibited a reduction of 17.8°C compared to the blank.

## Graphical abstract

**Figure:**
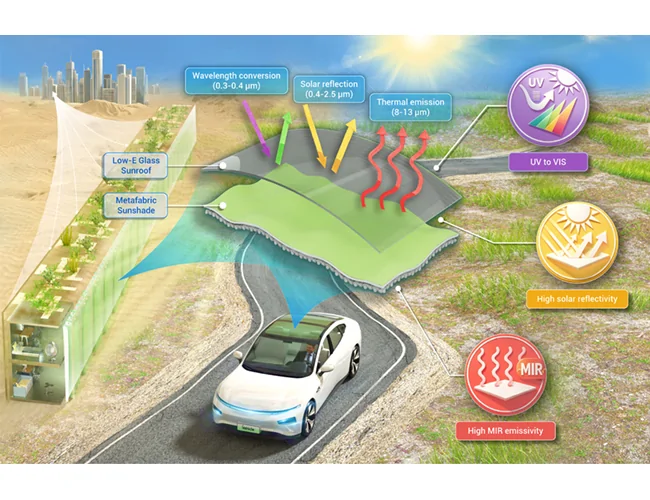

## Introduction

Global urbanization is accelerating, with the urban population projected to surge from 57% in 2022 to 68% by 2050.^1^ This rapid demographic shift, coupled with the proliferation of high-rise infrastructure and electric vehicles (EVs), exacerbates human exposure to the urban heat island (UHI) effect, intensifying the risk of thermal extremes.^2^^,^^3^^,^^4^ In modern commercialized urban areas, the dominance of glass-intensive high-rise offices and panoramic-sunroof EVs has redefined the built environment’s thermal interface. Therefore, mitigating the UHI effect’s impact on human health in high-rise office buildings and EVs is imperative and demands immediate attention from contemporary society.^5^^,^^6^^,^^7^^,^^8^^,^^9^ In the automotive industry, while low-emissivity (low-E) glass has been widely adopted to enhance cabin aesthetics and illumination,^10^ the intensifying global climate crisis has rendered conventional glazing inadequate. The resulting heat from the sun passing through the glass accumulation poses severe threats to passenger safety and triggers the premature aging of sophisticated vehicle polymers^11^—a challenge mirrored in glass-curtain high-rise offices. Air conditioning systems can effectively reduce the temperature inside a vehicle. However, they consume a significant amount of energy and do not protect against sun damage to occupants and interior components. Furthermore, commercial sunshades can insulate vehicles against ambient heating, but their weak thermal radiation insulation capability allows solar radiation to penetrate and heat the vehicle cabins. Hence, the thermal management of enclosed spaces behind glass urgently necessitates a paradigm shift toward more sustainable, passive strategies.

Passive radiative cooling (PRC) emerges as a transformative, zero-energy technology that utilizes precision micro/nano-optical engineering to modulate electromagnetic waves across solar and thermal bands.^12^^,^^13^^,^^14^^,^^15^^,^^16^^,^^17^ By tailoring the refractive index at multiple material interfaces, these structures manipulate photon propagation to suppress solar heating while facilitating thermal emission into the cold universe.^18^^,^^19^^,^^20^^,^^21^^,^^22^^,^^23^^,^^24^^,^^25^^,^^26^ Despite the proliferation of hierarchical designs—including hierarchical micro/nano-structural design,^27^^,^^28^ such as typical film categories consist of micro/nano-fiber films,^29^^,^^30^^,^^31^^,^^32^ metal reflection-polymer-dielectric composition multilayer films,^14^^,^^33^^,^^34^^,^^35^ porous films,^36^^,^^37^^,^^38^^,^^39^ photonic structure metamaterial multilayer films,^40^^,^^41^ and hierarchical-morphology metafabrics^42^—implementing these technologies within low-E glass systems remains a formidable challenge. The panoramic low-E glass sunroof exhibits high transmittance in solar wavelengths, particularly in terms of ultraviolet (UV) radiation and visible spectra (Figure S1A). Furthermore, low-E glass sunroofs exhibit absorption within the mid-infrared (MIR) radiation band (Figure S1B; Table S1), leading to heat accumulation inside the vehicle. Crucially, the inherently low emissivity of low-E coatings impedes the radiative dissipation of trapped interior heat, creating a feedback loop of elevated temperatures and material degradation. Additionally, the reliance on noble metals such as silver (Ag) and complex fabrication processes hinders large-scale industrialization,^43^^,^^44^ while excessive solar reflectivity risks causing hazardous white light pollution.^45^ Addressing these multifaceted constraints requires a fundamental innovation in optical structural design—balancing peak solar reflectance with minimized light pollution and high UV-aging resistance.

Here, we present a photoluminescent-assisted passive cooling strategy with hierarchical structure for post-glass spectral regulation inside high-rise office buildings and EVs, achieving significant cooling performance in real test scenarios (Figure 1A; Video S1). By integrating photoluminescence with radiative cooling, this combined strategy imparts unique spectrally selective properties to the material, significantly enhancing the cooling efficacy of the developed metafabric sunshade. The metafabric sunshade consists of a high MIR emission layer, an efficient solar radiation reflection layer, and a protection layer with enhanced transmission or emission in the MIR region. Grounded in Kirchhoff’s law of thermal radiation and electromagnetic theory, the material’s robust imaginary refractive index within the MIR spectrum facilitates intense thermal emission (Figure S2A). Furthermore, the fluorescent particles (FPs) embedded in the emission layer demonstrate exceptional photoluminescent capabilities, efficiently down-converting incident UV radiation into visible light that is re-emitted into the environment. The metafabric sunshade provides a sophisticated solution for energy-efficient cooling of glass rooms.

**Figure 1:**
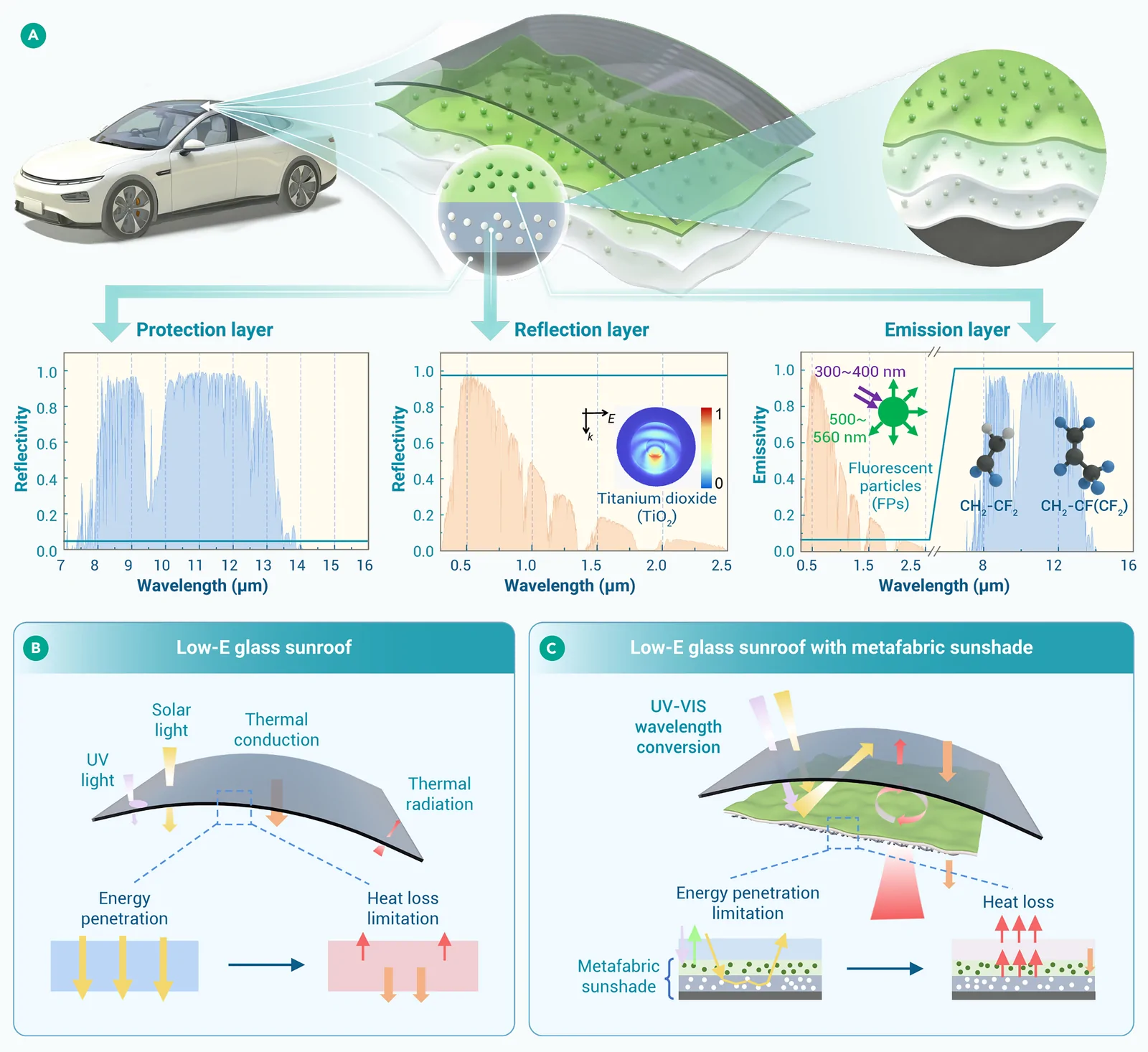
A photoluminescent-assisted scalable metafabric sunshade for significantly improved optical performance (A) Diagram of photoluminescent-assisted scalable passive cooling metafabric sunshade with broad spectrum modulation and micro/nano-optical structure design. (B) Radiant heat transfer mechanism of low-E glass sunroof. (C) Radiant heat transfer mechanism of low-E glass sunroof with metafabric sunshade.

Video S1. Overview movie Movie display of Photoluminescent-assisted Scalable Passive Cooling Hierarchical Metafabric Sunshade for sunburnt glass rooms and vehicles in extreme heat temperature. The movie demonstrates the scientific analysis of internal temperatures in glass rooms and vehicles equipped with low-E large sunroofs under extreme heat conditions. Furthermore, it highlights the research significance and innovation of our study aimed at achieving efficient passive cooling of the interior through hierarchical and photoluminescent metafabric sunshade. Cooling tests demonstrated that the metafabric sunshade in the vehicle interior resulted in a significant temperature reduction of 17.8° and 14.4° compared to the vehicles with blank and commercial sunshade, respectively. Moreover, this breakthrough in metafabric sunshade technology exhibits significant potential for enhancing passive thermal management applications such as glass rooms, vehicles, and outdoor shelters.

To maximize cooling power, the emission layer is optimized to minimize parasitic absorption within the solar band, effectively suppressing heat gain. Remarkably, our proposed reflection layer could reflect solar radiation efficiently through a well-designed combination of micro/nano-optical structures made by distinct materials. Meanwhile, the introduction of micro/nano-optical particles at the wavelength scale induces multiple Mie-type resonances toward solar radiation, while a greater difference (Δ*n*) in the refractive index enhances the scattering ability of the reflection layer toward solar radiation.^46^ This ultimately results in an elevated reflectivity rate (Figure S2B). Consequently, as solar radiation penetrates the reflective layer, it undergoes effective scattering that continuously alters its propagation direction; ultimately, the radiation deviates from its original incident trajectory and is reflected away from the metafabric sunshade. The protection layer is essential for maintaining optimal performance. The protection layer is equally vital, maintaining systemic integrity while ensuring high MIR transmittance or absorption to facilitate the efficient radiative transfer of interior heat to the external cold sink (Figure S2C). In a standard configuration, a low-E glass sunroof transmits substantial solar flux and traps MIR radiation, leading to a rapid rise in cabin temperature (Figure 1B). Conversely, the FP-enhanced metafabric absorbs deleterious UV radiation and re-radiates it as visible light. Even under peak solar irradiance, the low thermal conductivity of the metafabric acts as a superior thermal barrier, effectively decoupling the interior environment from the external thermal load (Figures 1C and S3).

## Materials and methods

### Materials

DMF (AR > 99.5%) and sodium dodecylbenzenesulfonate (SDBS; AR > 88%) were purchased from Sinopharm Chemical Reagent. P(Vdf-HFP) (PVDF 2801, 10% HFP) was purchased from Arkema. TiO_2_ (W400) was obtained from Venator Materials PLC. FPs (MHG, a pigment [SrAl_2_O_4_: Eu^2+^,Dy^3+^,Yb^3+^] with yellow-green luminescence) were obtained from Shandong Yousuo Chemical Technology. Nylon fabric was obtained from Guangzhou Pampas Trading. All other solvents and reagents were of analytical grade and were used as received.

### Fabrication of the metafabric sunshade

Firstly, the reflection layer of the metafabric sunshade was prepared by stirring P(VdF-HFP) powder in DMF solvent at a 1:12 ratio at 55°C for 30 min to obtain the P(VdF-HFP)-DMF co-mingled solution. The mass of titanium dioxide particles was determined based on weighing and calculating a 25% volume fraction of TiO_2_ in P(VdF-HFP). Additionally, a 1–2 wt % sodium SDBS mass of TiO_2_ was added to the P(VdF-HFP)-DMF co-mingled solution. The mixture was stirred at 55°C for 2 h to achieve the desired. The resulting solution of the reflection layer was subjected to sonication for a duration of 20 min. The scraping process was conducted at a room temperature of 25°C and a relative humidity of 80%. A scraper-abrasive knife, set to a thickness of 100 μm, was employed initially. Subsequently, after the film had dried and cured, further scraping was performed until a thickness of 300 μm was reached, which is the metafabric sunshade reflection layer. This sequential approach aimed to prevent the settling of titanium dioxide particles caused by excessively thick scraping in a single step. Lastly, there is the preparation of the fluorescent emission layer. Similarly, the P(VdF-HFP)-DMF co-mingled solution was first prepared. A FP mass of 70–80 wt % P(VdF-HFP) and a dispersant mass of 1–2 wt % FP were added to the P(VdF-HFP)-DMF co-mingled solution and stirred for 2 h to obtain the metafabric sunshade fluorescent emission layer. The resulting solution of the fluorescent emission layer was subjected to sonication for 20 min. The fluorescent emission layer was revised by employing a scraper with a precisely adjusted thickness of 40 μm. After allowing sufficient time for the fluorescence emission layer to dry, a metafabric sunshade was successfully obtained.

### Spectral characterization of the metafabric sunshade

The spectral properties of the metafabric sunshade were characterized separately in the solar spectrum (0.3–2.5 μm) and MIR (2.5–25 μm) wavebands. In the first range, the solar reflection and transmission spectra were recorded using a UV-visible-near-infrared (NIR) spectrophotometer (SolidSpec-3700) equipped with an integrating sphere model that used PTFE as the baseline material. For the second range, a Fourier transform infrared (FTIR) spectrometer (Nicolet iS50R) equipped with a gold integrating sphere was used to measure the absorption-emission and transmission spectra (gold was used as the baseline material).

### Morphology characterization of the metafabric sunshade

Optical images of the samples were taken by a digital camera (Nikon DSLR D5100). The microstructure of the metafabric sunshade was characterized by a scanning electron microscope (JSM7900F).

### UV radiation aging test of the metafabric sunshade

The durability of our metafabric sunshade was validated in a UV aging box (HS-UV-1300) at 62°C, where the metafabric sunshade was exposed to 340 nm UV light with a density of 1.2 W/m^2^ for 0, 24, 48, 72, 96, 120, 144, and 168 h, according to ASTM G-154 standards. The solar reflections of the metafabric sunshade were 94.3%, 93.62%, 94.11%, 93.44%, 94.94%, 92.44%, 92.48%, and 93.34%, respectively, and the change remained within 1.85% during the testing period. The solar reflections of the low-E glass sunroof and metafabric sunshade were 37.43%, 37.44%, 36.52%, 36.86%, 37.31%, 36.81%, 37.09%, 37.03%, respectively, and the change remained within 0.91% during the testing period. To assess long-term durability, accelerated aging tests were conducted in strict accordance with the GB/T 31899 standard. The cyclic aging test consisted of 36 consecutive cycles, with each cycle involving 8 h of UV irradiation and a subsequent 4 h of water spraying under a constant temperature of 60°C. After cumulative exposure to 288 h of UV radiation and 144 h of high-humidity water spraying, the metafabric sunshade retained a solar reflectivity of 90.0%.

### Friction stability

To assess the surface durability of the metafabric sunshade, friction resistance tests were performed in accordance with the GB/T 21196.2–2007 standard. Test specimens of the metafabric were secured on the test platform, and a friction head (loaded with a specified weight) was operated to rub the sample surface repeatedly at a controlled speed. After 100 consecutive friction cycles, the solar reflectivity of the post-test metafabric was characterized across the 0.3–2.5 μm wavelength range.

### Tensile performance

Tensile tests were conducted using a universal testing machine. The test specimens were cut into a standard size of 5 × 10 cm (width × length), and the tensile process was carried out at a constant speed of 2 cm/min. During the test, the tensile force and corresponding displacement of the samples (including nylon fabric and the metafabric sunshade) were continuously recorded, and the resulting force-displacement curves directly reflect the mechanical response characteristics of the materials under tensile loading.

### The thermal conductivity test of the metafabric sunshade

The thermal conductivity was measured with a thermal conductivity tester (TC3000E, Xi’an Xiatech Electronics) using the principle of the transient hot-wire method, and the average value was calculated from 5 measurements for each sample.

### Thermal measurements of the metafabric sunshade

The cooling effect of the metafabric sunshade applied to a glass room and the interior of a vehicle was measured. We built three glass rooms with five identical glass sizes and used 5-cm-thick insulating foam for the bases. In addition, we encapsulated the junction between the glass and the insulation foam to prevent internal and external heat transfer from affecting the test results. The glass rooms were divided into those with a metafabric sunshade attached, those with a metafabric sunshade without FPs, and those without any treatment for cooling experiments. On this basis, K-type thermocouples were mounted on the top of the glass room with the top and bottom sides of the glass room interior. The second test is the cooling experiment for vehicles, where we rented three vehicles that are basically the same in terms of vehicle model, color, year of production, and age for thermal measurements. One of the vehicles was treated by fixing the metafabric sunshade at the top of the sunroof inside, one was covered with a commercial sunshade, and the other vehicle was left untreated. On this basis, we used type K thermocouples to measure real-time temperature variations on the outer surface of the sunroof, the inner surface of the sunroof/lower surface of the metafabric sunshade, the upper side of the interior space of the vehicle, the lower side of the interior space of the vehicle, and the driver’s seat cushion and backrest. K-type thermocouples (TT-K-30-SLE) connected to a recorder (TC-08, Pico Technology) were used to monitor the sample’s temperature in real time. The thermocouples were carefully calibrated with a test error of 0.4°C for the outdoor tests and 0.1°C for the indoor measurements. Ambient temperatures for all thermal measurements are obtained from type K thermocouple tests. The solar irradiation intensity at the time of the test was obtained from the official Solcast website, a professional solar energy company.

### Water contact angle test of the metafabric sunshade

The water contact angle of the metafabric sunshade was tested using a Dataphysics OCA 20 instrument.

### Optical simulation calculations of the metafabric sunshade

Using numerical simulation software combined with optical simulation software, a complete modeling system of the structure of the metafabric sunshade was carried out, and perfect boundary conditions and scattering boundary conditions were added to construct a physical environment without external interference for the metafabric sunshade. In addition, in order to simultaneously satisfy the requirements of saving computational cost and effective theory-guided experiments, the width of the metafabric sunshade was chosen to be 15 μm during the simulation process. On this basis, we set the boundary conditions on both sides as periodic boundary conditions, and the electromagnetic wave transmitting and receiving ports were also set to periodic boundary conditions to satisfy the continuity at the physical boundaries. Ultimately, the ratio of the emitting port to the receiving port of the electromagnetic wave was calculated, and the required reflectance and transmittance were obtained. Regarding the absorption of UV radiation and MIR radiation, the energy absorbed/lost by the metafabric sunshade was obtained by calculating the electromagnetic loss integral of the metafabric sunshade when the electromagnetic wave propagates through the metafabric sunshade, and the emissivity/absorptivity was obtained from the final analysis and calculation.

## Results and discussion

### Hierarchical structure design

We constructed high-fidelity models of micro/nano-scale photonic structures based on the metafabric sunshade depicted in Figure 1A and conducted optical numerical simulations to characterize the solar band and MIR band.^47^ To optimize the radiative cooling potential, we systematically investigated the thickness-dependent emissivity of the P(VdF-HFP) matrix within the 0.3–20 μm range. Our simulations reveal that as the thickness approaches a 100 μm threshold, the average emissivity within the infrared atmospheric window converges to approximately 95% (Figures 2A and 2B; supplemental information). Furthermore, we are particularly interested in examining the transmittance of the emission layer in the solar band. The high transmittance in the solar band is attributed to the negligible imaginary part of the refractive index in most solar bands for P(VdF-HFP), resulting in minimal electromagnetic loss (Figure 2C).^48^ Meanwhile, the polymer features multiple extinction peaks in the thermal wavelength range, with coverage extending to the long-wavelength MIR window. These peaks originate from the diverse vibrational modes inherent to its molecular structure, thereby imparting outstanding emissivity to the polymer in the MIR region.^49^ A nuanced decrease in transmittance was observed in the NIR region (2.2–2.5 μm) as thickness increased. Although the imaginary refractive index remains relatively low in this specific NIR band, the cumulative absorption effect amplified by the increased optical path length eventually outweighs the low intrinsic loss, leading to the observed attenuation in transmittance.

**Figure 2:**
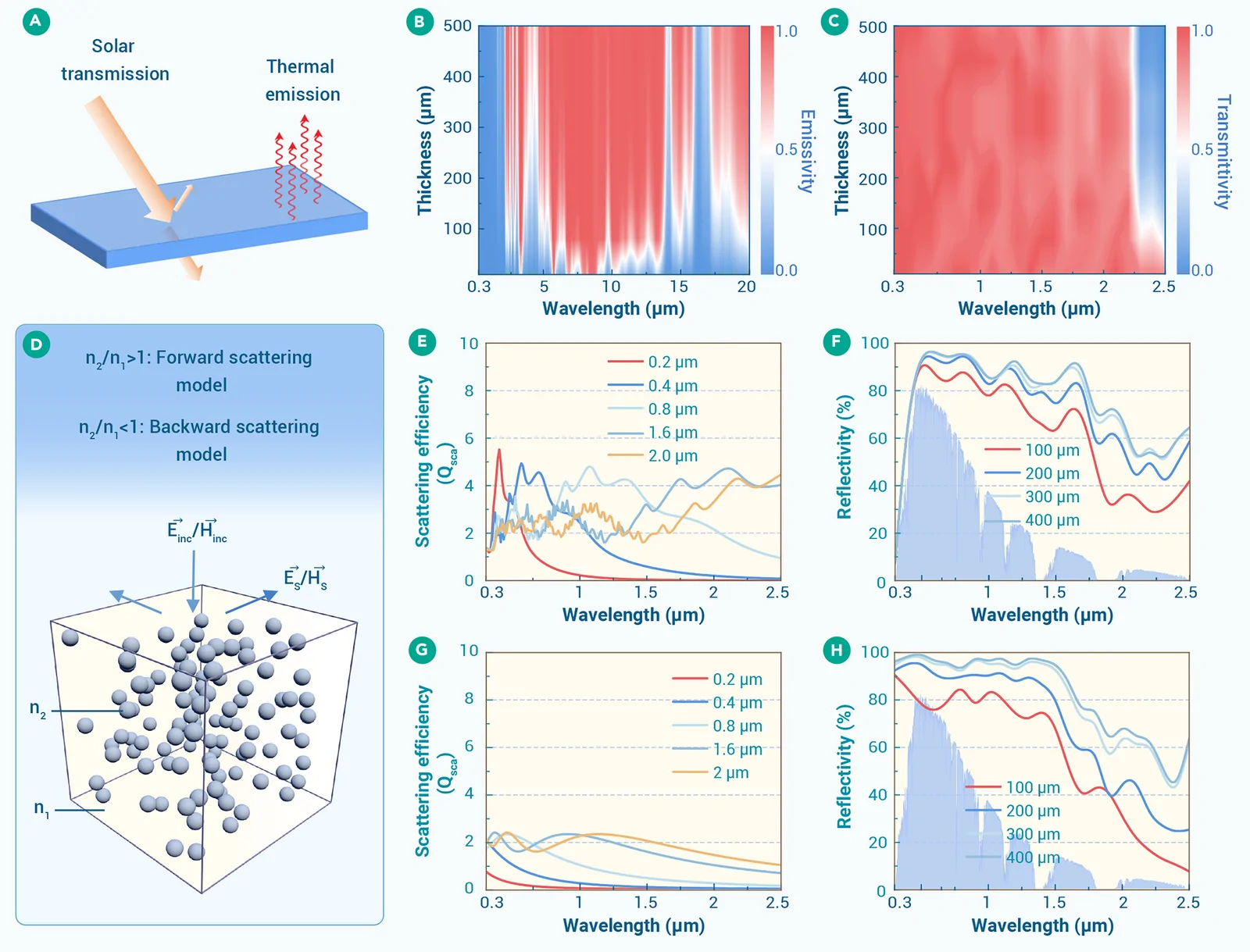
Optical simulation and optical parameter analysis of metafabric sunshade (A) Performance analysis of polymer-based materials meeting the requirements of the refractive index imaginary value. (B) Relationship between emissivity and thickness of the emission layer in the full spectral band. (C) The relationship between transmittance and thickness of the emission layer in the solar band. (D) Multi-physics field simulation model for metafabric sunshade. (E) Scattering efficiency of inorganic particles in the solar band. (F) Relationship between reflectivity and thickness of micro/nano-optical structures containing metal oxides. (G) Scattering efficiency of hollow cavities in the solar band. (H) Relationship between reflectivity and thickness of micro/nano-optical structures containing hollow cavities.

In addition to the excellent MIR emission capability exhibited by the emission layer, minimizing solar absorption is critical for high-performance passive cooling devices. The refractive index of the base material within the reflection layer is denoted as *n*_1_, while *n*_2_ represents the real parts of the refractive index for the micro/nano-photonic spheres. The interfaces between materials with different refractive indices can enhance and manipulate photon propagation direction, concurrently inducing Mie-type scattering on a wavelength scale.^50^ Hence, a relative refractive index *n*_rel_ = *n*_2_/*n*_1_ > 1 (forward scattering model) promotes forward-directed lobes, while *n*_rel_ < 1 (backward scattering model) shifts lobes oppositely, as governed by Mie theory under fixed X = 4.19 (X = 2πr/λ, with particle size r = 400 nm, incident electromagnetic wave λ = 600 nm; Figure 2D). In this study, we selected TiO_2_ (*n*_2_) as the micro/nano-photonic sphere because it possesses a high real part of the refractive index, and P(VDF-HFP) (*n*_1_) with a high imaginary part of the refractive index in the MIR band was selected as the base material for the reflection layer. The scattered intensity of the electromagnetic field is *E*_*s*_/*H*_*s*_ when a plane-wave electromagnetic field (*E*_*inc*_/*H*_*inc*_) is incident in the transverse electric and transverse magnetic modes. Numerical simulation results demonstrate the scattering efficiency of TiO_2_ particles within the solar band, and the scattering efficiency distribution at the corresponding wavelength is strongly correlated with the particle size (Figure S4A). The scattering efficiency of TiO_2_ particles with a size of 400 nm is the highest within the solar band compared to other sizes of TiO_2_ particles. When using a reflection layer thickness of 300 μm and a volume fraction of 25%, the weighted reflectivity in the solar band remains close to ∼93%, effectively mitigating emitter heating caused by solar radiation despite TiO_2_ absorption in the UV band (Figures 2E, 2F, S4B, and S4D).^51^ Furthermore, the optical properties of another micro/nano-photonic sphere (*n*_2_), composed of P(VdF-HFP) with a refractive index lower than TiO_2_, were numerically simulated (Figures 2G, 2H, and S5). Notably, regardless of whether TiO_2_ or micro/nano-cavities are used as particles in the reflection layer, the optimal reflectivity is consistently achieved at a thickness of 300 μm. In summary, precise spectral tuning can be achieved by synergistically optimizing the refractive index, particle size, volume fraction, and thickness.

### Material design and characterization

We fabricated the metafabric sunshade using a scalable knife-coating method, ensuring its potential for large-scale industrial application (Figure 3A; Video S2). The reflection layer was composed of randomly distributed rutile TiO_2_ particles exhibiting a precisely controlled normal distribution—within a P(VdF-HFP) matrix (Figures S6A and S7). Moreover, nylon fabric was used as the protection layer due to its strong MIR emissivity and adsorption properties. While TiO_2_ particles demonstrate outstanding solar reflectivity, their intrinsic absorption in the UV band elevates the metafabric sunshade temperature and impacts the cooling effect. To achieve precise regulation of the photons in the UV band, we incorporated randomly distributed FPs into the emission layer. These FPs possess high photoluminescent quantum yield, enabling them to capture high-energy UV photons and down-convert them into visible light, which is subsequently re-emitted into the environment (Figure S6B). This approach effectively mitigated the heating effects caused by UV radiation on both metafabric sunshades. Hence, placing the fluorescent material on the emission layer of the metafabric sunshade prevented UV radiation from penetrating into this layer. The resulting photoluminescent metafabric was produced with a width of 1.2 m, comprising an FP-doped emission layer (∼40 μm), a reflection layer (∼300 μm), and a nylon protection layer (∼60 μm) (Figures 3A, 3B, and S8). Scanning electron microscopy (SEM) characterization confirmed the well-defined hierarchical optical structure and demonstrated the highly homogeneous dispersion of TiO_2_ particles within the reflection layer.

**Figure 3:**
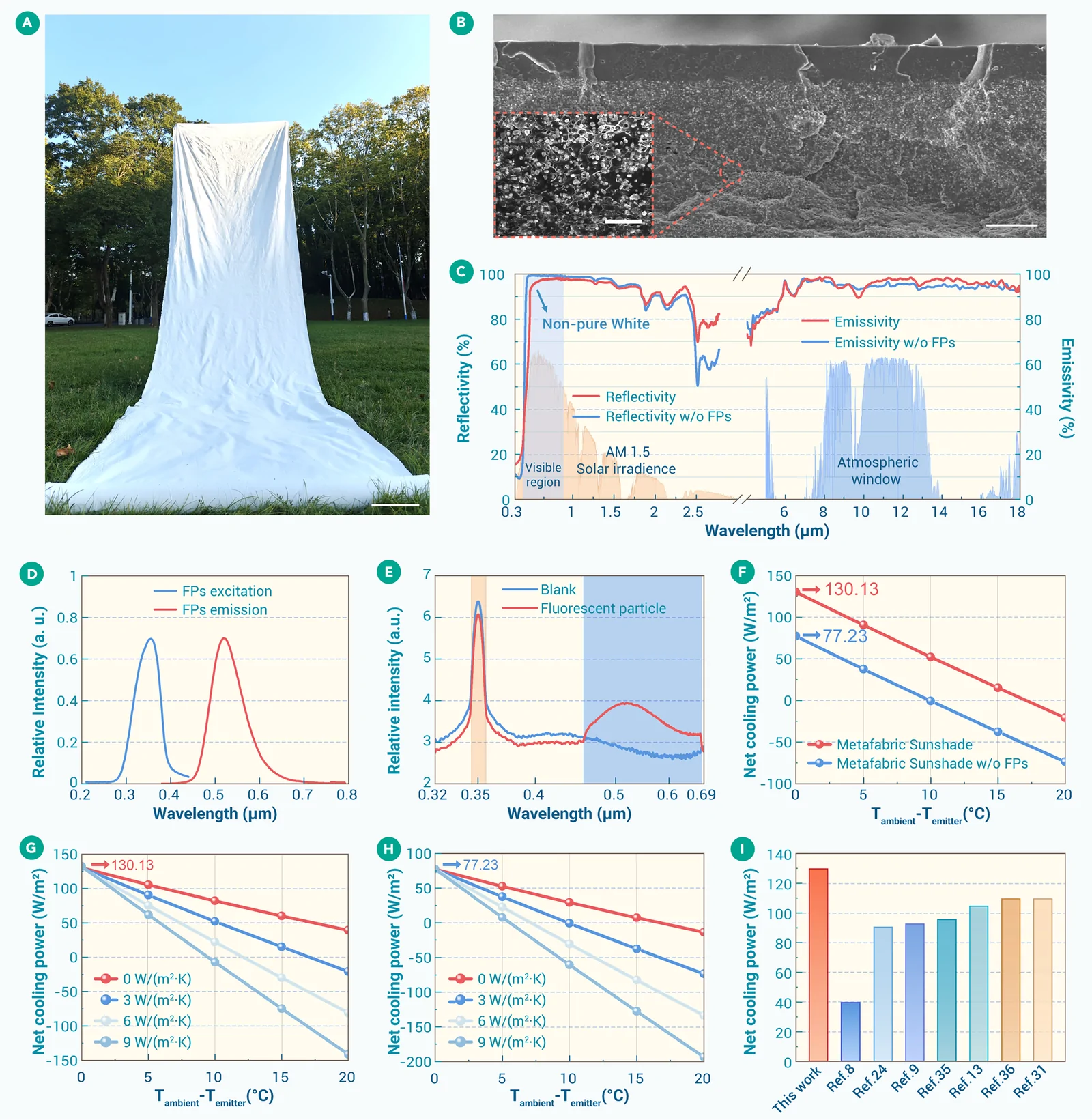
The photoluminescent metafabric sunshade is produced using a scalable squeegee coating process, characterizing the performances (A) The metafabric sunshade sample with a width of 1.2 m is displayed. Scale bar: 20 cm. (B) SEM image of metafabric sunshade with graded structure and SEM of uniformly distributed TiO_2_ particles in the reflection layer. Scale bar: 100 and 10 μm (inset). (C) Reflectivity in the solar band and emissivity in the MIR band for the metafabric sunshade and the metafabric sunshade without FPs. (D) Fluorescent particles have a normal-like absorption spectrum at 300–400 nm and are emitted as visible light. (E) Quantum yield calculations for fluorescent particles. (F) The calculated cooling power of the metafabric sunshade and the metafabric sunshade without FPs at ambient temperature (30°C) during the daytime (nonradiative heat transfer coefficients [h_c_] of 3 W/(m^2^·K) are used in the calculations). (G) The calculated cooling power of the metafabric sunshade at ambient temperature (30°C) during the daytime (nonradiative heat transfer coefficients [h_c_] of 0, 3, 6, and 9 W/(m^2^·K) are used in the calculations). (H) The calculated cooling power of the metafabric sunshade without FPs at ambient temperature (30°C) during the daytime (nonradiative heat transfer coefficients [h_c_] of 0, 3, 6, and 9 W/(m^2^·K) are used in the calculations). (I) A comparison between the metafabric sunshade and other materials reported in previous studies.

Video S2. The scalable scraping process of metafabric sunshade

The solar reflectivity of the metafabric sunshade was 94.3%, while its emissivity in the MIR band reached 95.8% (Figure 3C). Compared with the metafabric sunshade without FPs, the metafabric sunshade exhibited a slightly greater reflectivity in the UV band due to the photoluminescent ability of FPs. The metafabric sunshade showed high photoluminescent efficiency in the UV band, effectively down-converting incident UV light into visible light re-emitted into the environment (Figure 3D), as the entire sample exhibits a non-pure-white appearance (Figure S9). We evaluated the absolute quantum yield of the FPs used in this study and determined a photon conversion efficiency of 16.4%, representing a robust efficiency for translating absorbed photon energy into radiative emission (Figure 3E; supplemental information). It is crucial to place the entire sample in a UV-free environment for 24 h before and during the quantum yield testing on FPs. While solar band reflectivity and MIR band emissivity provide a foundational characterization of the metafabric sunshades’ optical performance, they are insufficient as sole evaluation criteria for cooling efficacy. The net cooling power must integrate complex environmental factors, such as solar radiation, atmospheric transmittance, and radiative cooling power, which effectively reflect the cooling performance of the metafabric sunshade under different nonradiative heat transfer coefficients (Figures 3F–3H).^52^^,^^53^ The maximum achievable net cooling power of non-photoluminescent metafabric was 77.23 W/m^2^ under isothermal conditions. However, due to the UV-to-visible conversion mechanism, conventional cooling power models fail to encompass the total energy flux; thus, the contribution of photoluminescence must be explicitly incorporated into the energy balance equation.^29^ Consequently, the optimized metafabric sunshade exhibited a net cooling power of 130.13 W/m^2^ (Figure 3F; supplemental information). The incorporation of photoluminescent functionality renders conventional optical metrics unable to accurately assess the cooling efficiency of the metafabric sunshade. While solar reflectance and emissivity remain critical, our work emphasizes the necessity of a holistic cooling power framework (supplemental information) to account for UV-to-visible conversion, aligning with established methodologies. Accordingly, in comparison to other studies on PRC, the metafabric sunshade demonstrates a substantial leap in performance in terms of net cooling power^13^^,^^14^^,^^24^^,^^35^^,^^42^^,^^49^^,^^54^ (Figure 3I).

The metafabric sunshade, both with and without FPs, showed high emissivity across the entire infrared spectrum, particularly in the MIR range. We benchmarked the spectral characteristics of bare sunroofs, metafabric sunshades, and commercial sunshades (Figure 4A; Table S1). To ensure a rigorous comparison, the selected commercial sunshades strictly comply with both ISO 13786 and GB/T 50189 standards, adhering to international benchmarks for optimal thermal performance. Based on the analysis of optical data, the metafabric sunshade exhibited significantly enhanced properties in terms of both reflectivity and emissivity compared to those of the sunshades (Figures 4B–4D). Furthermore, we optically characterized composite architectures comprising both sunroofs and sunshades. Compared with other combinations, the reflectivity of the sunroof-metafabric sunshade assembly exhibited superior performance, despite the thickness ratio of approximately 17.6 between the sunroof and the metafabric sunshade (Figures 4E–4G). Critically, the photoluminescent properties within the UV band synergistically augmented the cooling capabilities of the metafabric sunshade. Moreover, the emissivity in the MIR band remained virtually identical across all combinations due to the uniform sunroof thickness of 6 mm. In terms of heat transport, the metafabric sunshade possesses an exceptionally low thermal conductivity of 0.059 W/(m·K), which is an order of magnitude lower than that of the sunroof (0.987 W/(m·K)). This stark contrast effectively creates a thermal barrier, obstructing heat flux from the sunroof and the ambient environment into the vehicle interior (Figure S10A). Furthermore, the metafabric sunshade has a water contact angle of 138.8° (Figure S10B). The inherent self-cleaning functionality preserves the spectral integrity of the emitter over extended periods, thereby facilitating long-term durability in practical applications. The metafabric sunshade data demonstrated high resistance to UV aging. After being subjected to continuous UV irradiation for 168 h,^55^ the solar reflectivity fluctuated by only 1.85%, confirming exceptional spectral resilience and photostability (Figure S11). To assess long-term durability, accelerated aging tests were conducted in strict accordance with the GB/T 31899 standard. After cumulative exposure to 288 h of UV radiation and 144 h of high-humidity water spraying, the metafabric sunshade retained a solar reflectivity of 90.0% (Figure S12). In addition, abrasion resistance tests were conducted in accordance with the GB/T 21196.2-2007 standard, and the metafabric sunshade retained a solar reflectivity of 93.6% after 100 friction cycles (Figure S13A). We demonstrated the superior practical durability of the metafabric sunshade compared to current commercial benchmarks. Our tensile testing (Figure S13B) reveals that the metafabric exhibits a peak breaking force of approximately 127 N and an elongation at break of 27 mm, significantly outperforming the standard commercial sunroof sunshade, which failed at 98 N and 22 mm. This represents an approximately 30% increase in tensile strength, ensuring that the material can easily withstand the mechanical stresses of repeated folding, stretching, and automated retraction in sunroof systems. The scalable fabrication process and low-cost raw materials suggest significant potential for metafabric sunshades to be seamlessly integrated into next-generation autonomous vehicle thermal management systems (Tables S3 and S4).

**Figure 4:**
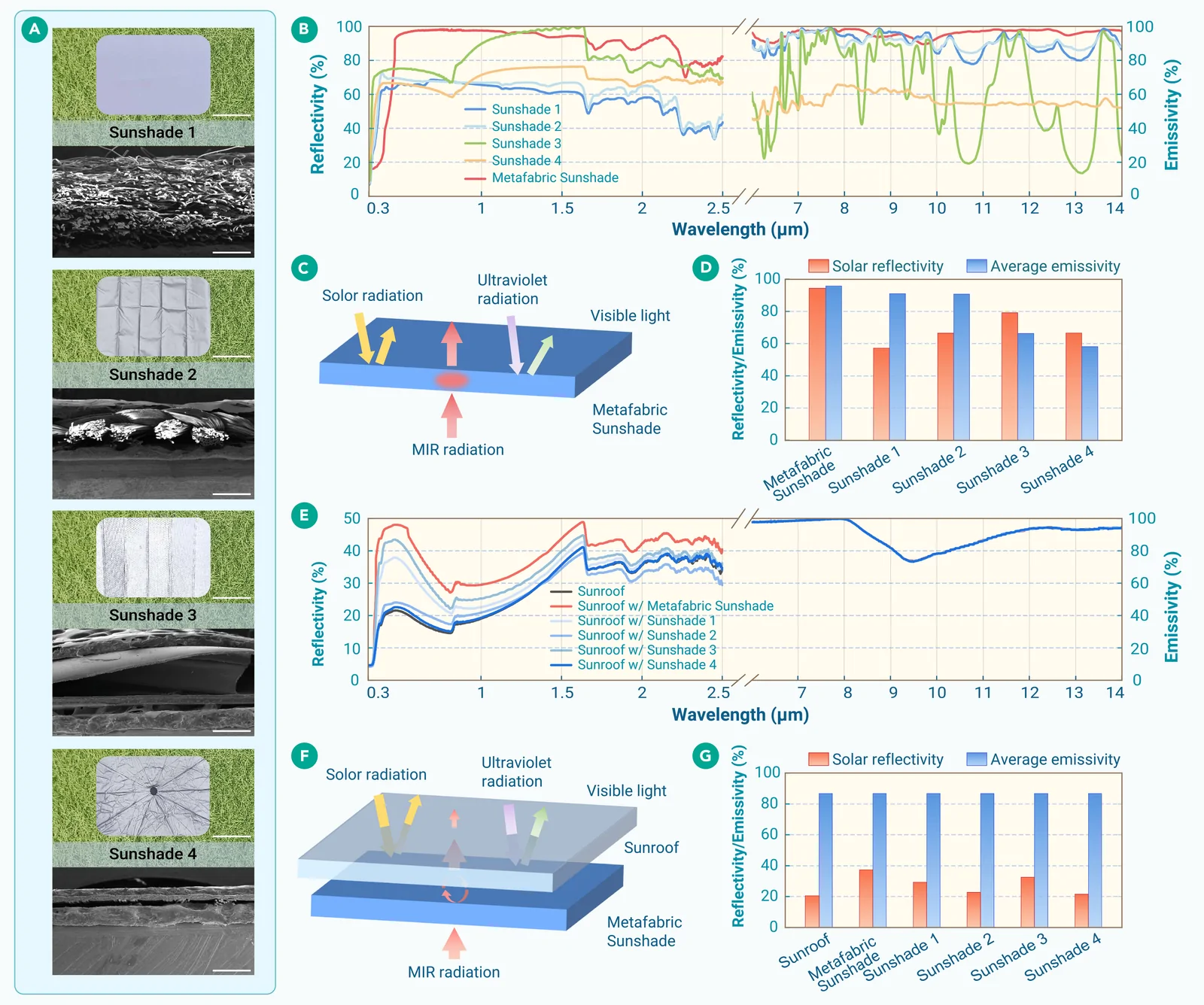
Comparison of optical properties and thermal conductivity of sunroof glass, sunshade, and metafabric sunshade (A) Physical and SEM images of commercial sunshades. Scale bar: 50 cm (top) and 100 μm (bottom). (B–D) Reflectivity and emissivity spectrum for sunroof, metafabric sunshade, and sunshades. Schematic diagram of passive cooling with the metafabric sunshade or a sunshade on the outer surface of the glass. Comparison of solar reflectivity and average emissivity spectra between the metafabric sunshade and sunshades. (E and F) Reflectivity and emissivity of glass (outside) and metafabric sunshade or sunshade (inside). Schematic of passive cooling when the metafabric sunshade or sunshade is on the inner surface of the glass. (G) Comparison of solar reflectivity and average emissivity spectra between glass, metafabric sunshade, and sunshades.

### Thermal measurements in the glass rooms and vehicles

In our field cooling experiments, the metafabric sunshade glass room exhibited significant cooling effects compared to the blank and conventional sunshade glass room, achieving maximum temperature reductions of 16.5°C and 9.6°C, respectively (Figures 5A–5C; supplemental information). During these outdoor evaluations, the metafabric sunshade demonstrated an average net radiative cooling performance of 154.35 W/(m^2^·K) (Table S5). The glass material used in glass rooms is high-transmission glass, which is commonly utilized in the construction industry (Figure S14). These empirical results validate the potency of the metafabric sunshade as an efficient passive cooling solution for glazing-centric architectural structures under extreme solar radiation. Furthermore, the contribution of FPs to the overall cooling performance was verified. Notably, the FPs integrated onto the metafabric surface displayed remarkable photoluminescent stability, resulting in a maximum decrease in temperature of 2.2°C compared to that of the metafabric sunshade without FPs (Figures S15 and S16).

**Figure 5:**
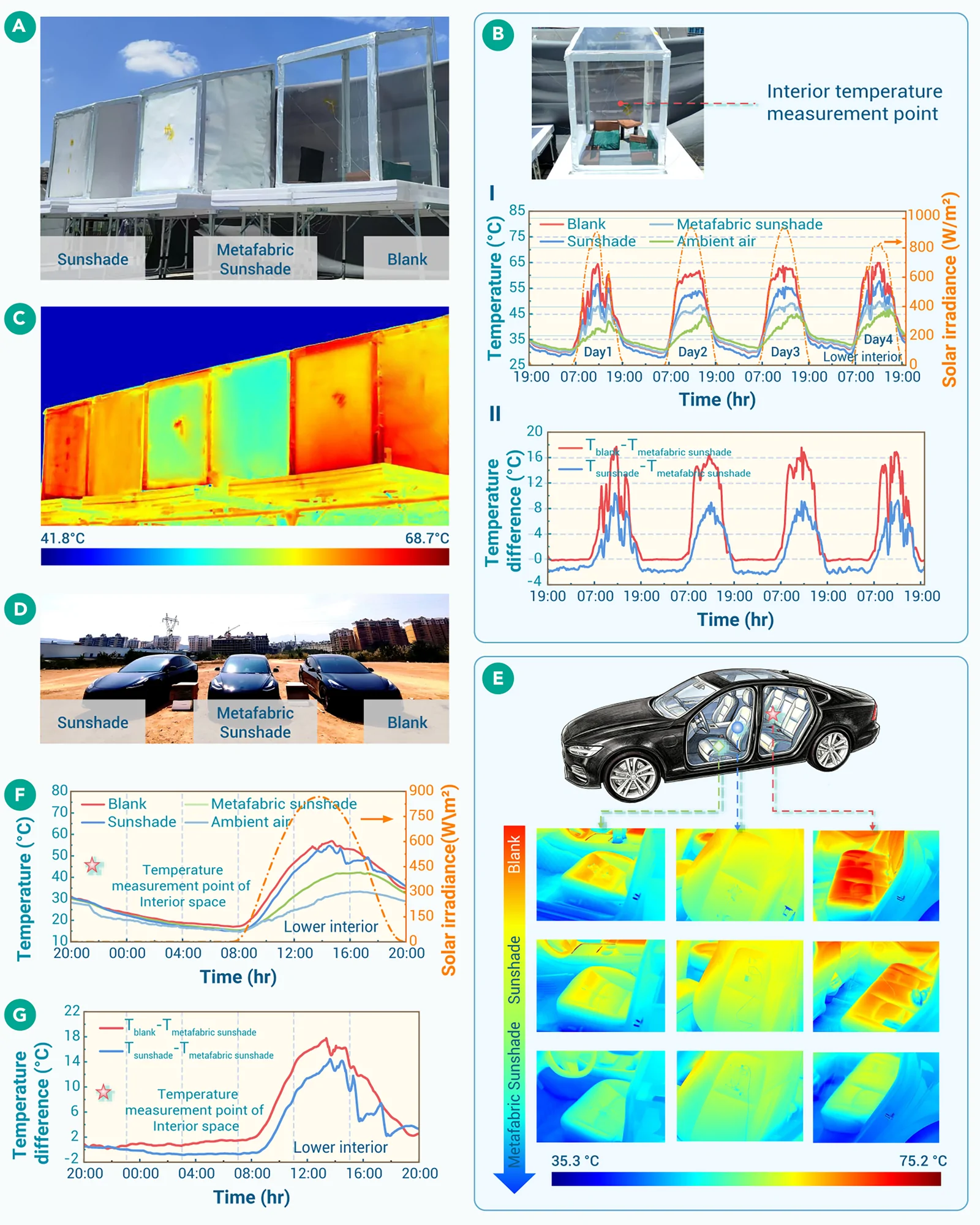
The metafabric sunshade was employed in cooling experiments conducted in a glass room and on vehicle sunroofs (A) Glass room cooling experiment diagram. (B-I) A 4-day continuous temperature measurement of the subambient cooling performance test in Wuhan, China (114°41Nʹ, 30°51′E, August 3–6, 2024). (B-II) The temperature difference between the glass without a cover, the metafabric sunshade, and the metafabric sunshade without FPs. (C) Infrared images of glass cooling experiments. (D) Physical drawings depicting cooling experiments on three vehicles, using a blank, a sunshade, and a metafabric sunshade, respectively. (E) Infrared images capturing the driver’s position and the rear seat in three vehicles. (F and G) Continuous 24-h temperature measurements obtained during the subambient cooling performance test conducted in Sipsongpanna, China (21°45′N, 100°36′E, February 22 and 23, 2024).

Building upon its exceptional PRC performance in architectural glazing experiments, the metafabric sunshade presents transformative potential for EV thermal management. Modern vehicles are increasingly equipped with expansive, optically transparent panoramic sunroofs. However, this design trend subjects the cabin to intense direct sunlight, leading to significant heat accumulation during operation. Critically, exposure to intense and high-energy UV radiation triggers the off-gassing of formaldehyde and other hazardous volatile organic compounds (VOCs) while simultaneously accelerating the photo-oxidative degradation of interior components and posing a substantial risk to passenger health. The metafabric sunshade addresses these challenges by synergizing high-efficiency solar reflection with selective thermal emission to the cold outer space. This work demonstrates a vehicular thermal management breakthrough by pioneering the integration of spectrally engineered metafabric sunshades, realizing a zero-energy sustainable cooling system. To validate this system in a real-world context, three identical vehicles (with the same model, color, and year of manufacture) equipped with a 1,340 × 1,050 mm panoramic sunroof were selected (Figure 5D). Comprehensive thermal profiling was performed through real-time monitoring of the temperatures at multiple critical nodes, including the sunroof interior surface, interior space (upper and lower sides), the driver’s seat surface, and the seat backrest (Figure 5E), and we also characterized the local atmospheric transmittance (Figure S17).

To evaluate real-world efficacy, three experimental groups were established: a vehicle equipped with the metafabric sunshade affixed to the sunroof’s interior interface, a control vehicle with a blank sunroof, and a third vehicle utilizing a standard commercial sunshade. All vehicles were facing north to ensure uniform solar exposure. Infrared thermography was employed to visualize the thermal distribution after prolonged exposure to intense solar radiation. Strikingly, significant cooling effects were observed in both the rear seat and the driver’s seat areas of the vehicle with the metafabric sunshade, compared to the blank and commercial counterparts. Moreover, the temperatures on the upper and lower sides of the interior in the blank vehicle and the vehicle with the metafabric sunshade were compared. Equipped with the metafabric sunshade, the vehicle exhibited a significant and consistent reduction in temperature on both the upper and lower interior, with a maximum decrease of 9.2°C and 17.8°C, respectively, compared to the blank vehicle, and 7.9°C and 14.4°C, respectively, relative to the vehicle with the commercial sunshade. (Figures 5F, 5G, and S18). During these field vehicle cooling evaluations, the metafabric sunshade demonstrated an average net radiative cooling performance of 459.69 W/(m^2^·K) (Table S6). Simultaneous real-time monitoring of the driver’s seat ergonomics showed that the backrest and cushion can be cooled by up to 5.4°C and 3.7°C, respectively, compared to the blank vehicle, and 4.8°C and 3.3°C, respectively, compared to the commercial sunshade (Figures S19 and S20). Beyond thermal management, the engineered metafabric harmonizes aesthetic versatility with sustainable cooling performance. The interior surface of the sunshade is a deterministic factor in shaping the cabin’s temperature gradient. Hence, the metafabric sunshades can reduce the temperature for the interior surface of the sunroof and commercial sunshade by up to approximately 11.6°C and 7.9°C, respectively, compared to vehicles with blank and commercial sunshades (Figure S21). Furthermore, the metafabric sunshade demonstrates efficient wavelength conversion when exposed to a UV lamp (Figure S22). Upon transitioning from the UV lamp to ambient energy-saving lighting, the photoluminescent properties of the metafabric provide a sophisticated, non-pure-white aesthetic (Videos S3 and S4). Our innovative work is expected to contribute significantly to the widespread proliferation of passive cooling metafabric, offering a transformative solution for the vehicle industry in heat situations.

Video S3. Demonstration of the photoluminescent and non-pure-white capability in metafabric sunshade

Video S4. Demonstration of the photoluminescent capability in metafabric sunshade

## Conclusion

In conclusion, we present a photoluminescent-assisted hierarchical metafabric sunshade designed for post-glass spectral regulation and highly efficient passive cooling. The industrial-scale production of these micro/nano-photonic structures can be accomplished using a simple and scalable squeegee coating process. The metafabric sunshade exhibits a high reflectance of 94.3% in the solar band and excellent emissivity of 95.8% in the MIR band. Critically, the integration of FPs significantly reduces the effects of UV radiation on metafabric sunshades by facilitating spectral down-conversion, thereby considerably improving the passive cooling performance of metafabric sunshades. This synergy significantly elevates the passive cooling performance beyond the limits of conventional radiative emitters. The metafabric sunshades demonstrate sustainable passive cooling performance in various outdoor applications and significantly outperform both sunroof and commercial sunshades in reducing vehicle interior temperatures. Beyond its optical prowess, the metafabric possesses multi-functional attributes, including intrinsic hydrophobicity, mechanical flexibility, enhanced UV shielding, and exceptionally low thermal conductivity. Thus, through efficient micro/nano-structural design and material optimization, metafabric sunshades are highly effective for sustainable passive cooling of the interiors of low-E glass sunroofs in vehicles exposed to extreme heat. Metafabric sunshades are highly promising for post-glass spectral regulation in high-rise buildings, vehicles, and various other fields. As global urbanization intensifies and the UHI effect presents escalating challenges, this micro/nano-optical regulation technology offers an effective solution to mitigate the associated impacts on human health and the environment. In addition, this work may have significant implications for future human exploration of the universe, where specific spectral regulation solutions will be crucial.

## Resource availability

### Materials availability

All materials used in this study are commercially available or can be synthesized according to the procedures described in the materials and methods.

### Data and code availability

All data are available in the main text or the supplemental information. Information requests should be directed to the corresponding authors.

## Funding and acknowledgments

This work was funded by the 10.13039/501100001809National Natural Science Foundation of China (grant nos. U2441275 and T2425018 to G.T.), Fundamental and Interdisciplinary Disciplines Breakthrough Plan of the 10.13039/501100002338Ministry of Education of China (grant no. JYB2025XDXM402 to G.T.), the Interdisciplinary Research Program of 10.13039/501100003172HUST (grant no. 2023JCYJ039 to G.T.), the National Natural Science Foundation of China (10.13039/501100001809NSFC) (grant no. 62222511 to Y.M.), STI 2030-Major Projects 2021ZD0200401, the 10.13039/501100004731Natural Science Foundation of Zhejiang Province China (grant no. LR22F050006 to Y.M.), the State Key Laboratory of Bio-based Fiber Materials (Grant No.SKLBFM202509 to X.F.), and the Innovation Fund of 10.13039/501100008416Wuhan National Laboratory for Optoelectronics (to G.T.). We are thankful for the support from the New Cornerstone Science Foundation through the XPLORER PRIZE; to Jiaxin Li, Maiping Yang, Yuqi Zou, and Pan Li for the support in the experiment section; to Chong Hou and Ning Zhou for support in the numerical simulations; and to engineer Wei Xu at the Optoelectronic Micro&Nano Fabrication and Characterizing Facility, Wuhan National Laboratory for Optoelectronics of Huazhong University of Science and Technology, for the support in device fabrication (spectral test). We thank engineer Tiantian Zeng at the Analytical & Testing Center, Huazhong University of Science and Technology, for the support in device fabrication (spectral test).

## Author contributions

G.T. proposed the project. Numerical simulations were carried out by M.L. and J.L. under the supervision of G.T. and C.-W.Q. Material batch preparation was done by M.C., S.Z., and J. Wu. Measurements and data evaluation were carried out by S.Z. and J.L. All authors contributed to the manuscript and approved the final version.

## Declaration of interests

The authors declare no conflicts of interest.

## Supplemental information

Document S1. Figures S1–S20, Tables S1–S16, and supplemental materials and methods

Document S2. Article plus supplemental information
