# Supplementary material for: Photoluminescent-assisted hierarchical metafabric sunshade for high-efficiency passive cooling
Source: Innovation (Camb). 2026 Jul 9;7(11):101503. doi: 10.1016/j.xinn.2026.101503 (PMC13751216; doi:10.1016/j.xinn.2026.101503)
Supplement: Supplementary file 1 [file mmc1.pdf]

The Innovation, Volume *7*

Supplemental Information

Photoluminescent-assisted hierarchical metafabric sunshade for high-efficiency passive cooling

Shiliang Zhang, Mingxiang Liu, Jingwen Wang, Mingyue Chen, Jiawei Wu, Jun Liang, Zitan Yang, Wei Wei, Yaoguang Ma, Cheng-Wei Qiu, Huamin Zhou, and Guangming Tao

This PDF file includes:

Supplementary information
Supplementary Figs. 1 to 22
Supplementary Tables 1 to 6
Supplementary References (1 to 7)

Other Supplementary Materials for this manuscript include the following:

Supplementary Videos 1 to 4

Supplementary information

Simulation model

The TiO_2 particles in the metafabric sunshade are modeled as spheres with radii, the radiation properties of the TiO_2 particles can be characterized by the scattering efficiency obtained from Mie scattering theory. The COMSOL Multiphysics (COMSOL) is a rigorous method of solving Maxwell's equations that have been used for the numerical simulation. To investigate the effect the different pore sizes have on metafabric sunshade optical properties, COMSOL is employed. To save computational time cost, two-dimensional models of circular pores were used. The scattering efficiency of different sized TiO_2 particles in the sun spectrum area (0.3-2.5 μm) was simulated using the total-field scattered-field source inside the Mie theory framework *(1)*.

Optical parameter analysis of metafarbic sunshade with photonic sphere

The optical properties of another micro/nano photonic sphere, composed of P (VdF-HFP) with a refractive index lower than that, were numerically simulated (Fig. 2D). Despite its ubiquity, air is a natural substance characterized by a refractive index of 1, rendering it suitable for utilization as a micro and nano photonic sphere with an exceptionally low real part of the refractive index (n_2). Given the distinct relative refractive indices of incident media in the electromagnetic field, it becomes evident that the micro/nano photonic sphere exhibiting a high real part of refractive index demonstrates superior capability in confining scattered electromagnetic waves compared to its counterpart, the micro/nano photonic sphere, characterized by a low real part of refractive index. In contrast, the micro and nano photonic sphere with a low refractive index real part (n_2) exhibits an extended forward scattering distance, facilitating the induction of multi-level electromagnetic wave resonance and ultimately enabling light to escape from the reflecting layer (fig. S4A). Moreover, the scattering efficiency of a single cavity is closely correlated with its radius, making 400 nm cavity optimal in comparison to other cavities within the solar radiation band (Fig. 2G). Furthermore, the concentration of microcavities in the substrate material significantly impacts the reflectivity exhibited by the metafabric sunshade in the solar radiation band, with an optimal concentration of 50% observed when investigating its reflectivity as a function of thickness (fig. S4B). Surprisingly, the reflectivity nearly reaches the threshold value at a thickness of 300 μm and concentration of 50%, and the influence of thickness is negligible (Fig. 2H, figs. S4, C to D). Unlike TiO_2 with its high refractive index, the microcavity's reflectivity approaches unity in the UV radiation band, effectively minimizing potential hazards from UV radiation. It is also noteworthy that regardless of TiO_2 or micro and nano cavities are used as particles in the reflection layer, optimal reflectivity is consistently achieved at a thickness of 300 μm. In summary, precise spectral tuning can be achieved in micro/nano photonic structured metafabric sunshades by satisfying requirements related to refractive index, particle size, concentration, and thickness.

Calculation and analysis of quantum yield for photoluminescence of fluorescent particles

Since fluorescent particles are unique optical particles, by absorbing the energy of electromagnetic waves in the UV band, they cause electrons to jump from a low energy level (usually the ground state) to a higher energy level (excited state). During energy absorption, electrons may return to lower energy states via non-radiative processes, such as internal conversion and intersystem crossing, or through radiative processes, such as fluorescence and phosphorescence. Fluorescence occurs when electrons in an excited state return to the ground state or another low-energy state by emitting photons. The emitted photons typically have a longer wavelength than the absorbed photons, a phenomenon known as the Stokes shift. This shift results from the loss of some electron energy in non-radiative forms, such as heat, before the electron returns to the ground state. The energy of a single photon is *hv*. The energy absorption that occurs during the photoluminescence of the fluorescent particles *(2)* is

$$E_{abs} = hv_{exc} - hv_{emi} \tag{1}$$

This is further calculated as

$$E_{abs} = \frac{hc}{\lambda_{exc}} - \frac{hc}{\lambda_{emi}}\varphi \tag{2}$$

Here, h is the the Planck constant, c is the speed of light in vacuum, λ_{exc} is the excited UV electromagnetic waves, $\lambda_{emi}$is the Electromagnetic waves emitted in the visible band, φ is the absolute quantum yield. The calculation of the absolute quantum yield requires the capture and acquisition of the number of excited photons (C_1) and the number of photons emitted by the fluorescent particles after their absorption and conversion (C_2) by the QuantaMaster 8000 steady-state transient modular fluorescence spectrometer. Consequently, φ can be calculated by the following equation.

$$\varphi = \frac{C_2}{C_1} \tag{3}$$

This is further calculated as

$$\varphi = \frac{\int_{345}^{356} N_{\lambda_{exc}} d\lambda_{exc}}{\int_{455}^{684} N_{\lambda_{emi}} d\lambda_{emi}} \tag{4}$$

Here, $N_{\lambda_{exc}}$ is the number of photons emitted by the instrument to excite the fluorescent particles, $N_{\lambda_{emi}}$ is the number of photons emitted by the fluorescent particle when it returns to the electronic ground state after absorbing the energy of the UV electromagnetic wave (Fig. 3E).

Calculation of cooling power

According to the law of Kirchhoff, the emissivity of an arbitrary body is equal to its absorptivity in thermodynamic equilibrium. So Emissivity ($\varepsilon(\lambda)$) was calculated by $\varepsilon(\lambda)$=1- R (λ)- T(λ) [3]. The average solar reflectance ($\bar{R}_{solar}$) is defined as *(3)* :

$$\bar{R}_{solar} = \frac{\int_{0.3\ \mu m}^{2.5\ \mu m} I_{solar}(\lambda) \cdot R_{solar}(\lambda) d\lambda}{\int_{0.3\ \mu m}^{2.5\ \mu m} I_{solar}(\lambda) d\lambda} \tag{5}$$

Where λ is the wavelength of the sunlight in the range of 0.3-2.5 µm, $I_{solar}(\lambda)$ is ASTM G173-03 Global solar intensity spectrum (AM1.5), and $R_{solar}(\lambda)$ is spectral reflectivity of the material. The average emittance ($\bar{\varepsilon}_{ATW}$) in the atmospheric transmittance window is defined as:

$$\bar{\varepsilon}_{ATW} = \frac{\int_{8\ \mu m}^{13\ \mu m} I_{BB}(\lambda)\cdot\varepsilon_{ATW}(\lambda)d\lambda}{\int_{8\ \mu m}^{13\ \mu m} I_{BB}(\lambda)d\lambda} \tag{6}$$

Where $\varepsilon_{ATW}(\lambda)$ represents spectral thermal emittance of the surface in the range from 8 µm to 13 µm. $I_{BB}(\lambda)$ represents the radiant intensity emitted by a blackbody at a temperature of T, and it can be calculated by the following equation:

$$I_{BB}(T,\lambda) = \frac{2hc^2}{\lambda^5}\frac{1}{e^{\frac{hc}{\lambda K_B T}}-1} \tag{7}$$

Where h is Planck's constant, K_B is the Boltzmann constant, and c is the speed of light.

The daytime cooling power of radiative cooler is calculated by the following equation:

$$P_{cooling} = P_{rad}(T) - P_{sun} - P_{atm}(T_{amb}) - P_{non-rad} \tag{8}$$

Where P_{rad} (T) represents the power radiated out by the radiative cooler at temperature T, which can be defined as:

$$P_{rad}(T) = 2\pi \int_0^{\frac{\pi}{2}} d\theta sin\theta cos\theta \int_{2.5\ \mu m}^{25\ \mu m} I_{BB}\ (T,\lambda)\ \varepsilon(\lambda,\theta)d\lambda \tag{9}$$

$I_{BB}(T, \lambda)$ is calculated by eq.(7), and $\varepsilon(\lambda, \theta)$ is the spectral and angular emissivity of the cooler. Atmospheric thermal radiation absorbed by the radiative cooler can be calculated as follows:

$$P_{atm}(T_{amb}) = 2\pi \int_0^{\frac{\pi}{2}} d\theta sin\theta cos\theta \int_{2.5\ \mu m}^{25\ \mu m} I_{BB}\ (T,\lambda)\ \varepsilon(\lambda,\theta)\varepsilon_{atm}(\lambda,\theta)d\lambda \tag{10}$$

$\varepsilon_{atm}(\lambda, \theta)$ is the spectral and angular emissivity of the atmosphere, which can be defined as $\varepsilon_{atm}(\lambda, \theta) = 1 - t(\lambda)^{1/\cos\theta}$. Here $t(\lambda)$ is the atmospheric transmittance in the zenith direction, which can be obtained by MODTRAN of Mid-Latitude Summer Atmosphere Model (MODTRAN® (spectral.com)) *(4, 5)*.

The solar irradiance absorbed by cooler (P_{sun}) and the non-radiative power lost ($P_{non-rad}$) due to convection and conduction is defined as:

$$P_{sun} = \int_{0.4\ \mu m}^{2.5\ \mu m} I_{solar}(\lambda)\ \varepsilon(\lambda,0)d\lambda \tag{11}$$

$$P_{non-rad} = h_c(T_{amb} - T) \tag{12}$$

Where h_c is the coefficient of the non-radiative heat.

The h_c involved in the actual experiment is calculated as follows：

We obtained weather data for the vehicle cooling experiment conducted in Xishuangbanna Dai Autonomous Prefecture, Yunnan Province, China, on 23 February 2024. Based on these data, we estimated certain parameters under conditions of a wind speed of 2.25 m/s, a relative humidity of 70% (Supplementary Fig. 13a), and an average ambient temperature of 29°C (measured via a Type K thermocouple).

Weather data were sourced from https://tianqi.eastday.com/tianqi/xishuangbanna/20240223/.

The convective heat transfer coefficient can be calculated from the Nusselt number (Nu), the Gerashov number (Gr), and the Prandtl number (Pr) *(6, 7)*:

$$Nu = 0.59 \cdot (Gr \cdot Pr)^{1/4} \quad (13)$$

$$h_{nature} = \frac{Nu \cdot \lambda}{L} \quad (14)$$

$$Gr = \frac{g \cdot \beta \cdot \Delta T \cdot L^3}{v^2} \quad (14)$$

The forced convection heat transfer coefficient can be calculated using empirical equations:

$$h_{forced} = 0.23 \cdot Re^{0.8} \cdot Pr^{0.4} \quad (15)$$

$$Re = \frac{U \cdot L}{v} \quad (16)$$

The heat transfer coefficients for natural and forced convection are vectorially superimposed:

$$h_c = \sqrt{h_{nature}^2 + h_{forced}^2} \quad (17)$$

In the glass room cooling experiment, we collected weather data from 3 to 6 August 2024 in Hongshan District, Wuhan, Hubei Province, China. The wind speed during this period was estimated to range from 1.6 to 3.3 m/s, and the relative humidity was documented at 81% (Supplementary Fig. 13). The average ambient temperature was measured as 37.4 ℃ using a Type K thermocouple.

Weather data were sourced from https://tianqi.eastday.com/tianqi/wuhan/20240803/.

Thermal measurements in glass room

The cooling effect of blank, sunshade, and metafabric sunshade in a glass room was tested using a precise temperature measuring device. To demonstrate the mass production of metafabric sunshade, we prepared five pieces of 1.5 m×1.2 m×340 μm metafabric sunshade and five pieces of sunshade, as well as three 1m×1m×1m glass room with an 8 mm thickness for cooling experiments (Fig. 5a). Prior to testing, each enclosure was placed on a 9 cm thick insulating foam board to isolate geothermal heat effects from the cooling experiment. Continuous temperature data were obtained by affixing blank, sunshade, and metafabric sunshades to the interior surfaces of the glasshouses during 96 hours of outdoor cooling experiments. In contrast to surface temperatures influenced by environmental factors, internal temperatures within the glasshouses showed a relatively stable longitudinal gradient. Therefore, we recorded lower side internal temperature data for all three sets of cooling experiments. The metafabric sunshade glass room exhibited significant cooling effects compared to the initial upper side interior portion of the blank and sunshade glass room in the cooling experiments, with maximum decreases in temperature of 16.5℃ and 9.6 ℃, respectively (Fig. 5b).

In addition, the contribution of FPs to real-world refrigeration experiments has been verified. The configuration is depicted in the figure, with the leftmost glass room representing the cooling sunshade devoid of fluorescent particles, the central denoting metafabric sunshade following the incorporation of fluorescent particles, and the right representing the blank control sample. The cooling effect of metafabric sunshade and metafabric sunshade without FPs in a glass enclosure was tested using a precise temperature measuring device. To demonstrate the mass production of metafabric sunshade, we prepared five pieces of 1.5 m×1.2 m×340 μm metafabric sunshade and five pieces of 1.5 m×1.2 m×300 μm metafabric sunshade without FPs each, as well as three

1m×1m×1m glass room with an 8 mm thickness for cooling experiments (Supplementary Fig. 10a). Prior to testing, each enclosure was placed on a 5cm thick insulating foam board to isolate geothermal heat effects from the cooling experiment. Continuously temperature data were obtained by affixing metafabric sunshade and metafabric sunshade without FPs to the exterior surfaces of the glasshouses after 12 hours of outdoor cooling experiments. In contrast to surface temperatures influenced by environmental factors, internal temperatures within the glasshouses showed a relatively stable longitudinal gradient. Therefore, we recorded lower side internal temperature data for all three sets of cooling experiments. Both the metafabric sunshade and metafabric sunshade without FPs glasshouses exhibited significant cooling effects compared to the initial upper side interior portion of the glass room in the cooling experiments, with maximum decreases in temperature of 16.6°C and 14.7°C, respectively (Supplementary Fig. 10b). Additionally, an significant temperature gradient inside the MS glasshouse where average lower side temperatures were found to be approximately 0.5 ℃ higher than upper side temperatures on average. This is attributed to heavier hot air masses presented on its interior upper side compared with colder air masses on its interior lower side. Thus, a more pronounced thermal gradient within this particular type of greenhouse structure was generated(Supplementary Fig. 10d). The expression in the manuscript has been modified accordingly. The results indicated that both metafabric sunshade and metafabric sunshade without FPs glasshouses achieved decreases in temperature up to 16.1 ℃ and 14.3 ℃, respectively, when compared to their respective initial lower internal portions (Supplementary Fig. 10e). Furthermore, it was expected that metafabric sunshade would exhibit a decrease in temperature by approximately 1.9 ℃ relative to its counterpart metafabric sunshade without FPs at its lower internal side portion as observed during experimentation (Supplementary Fig. 10f).

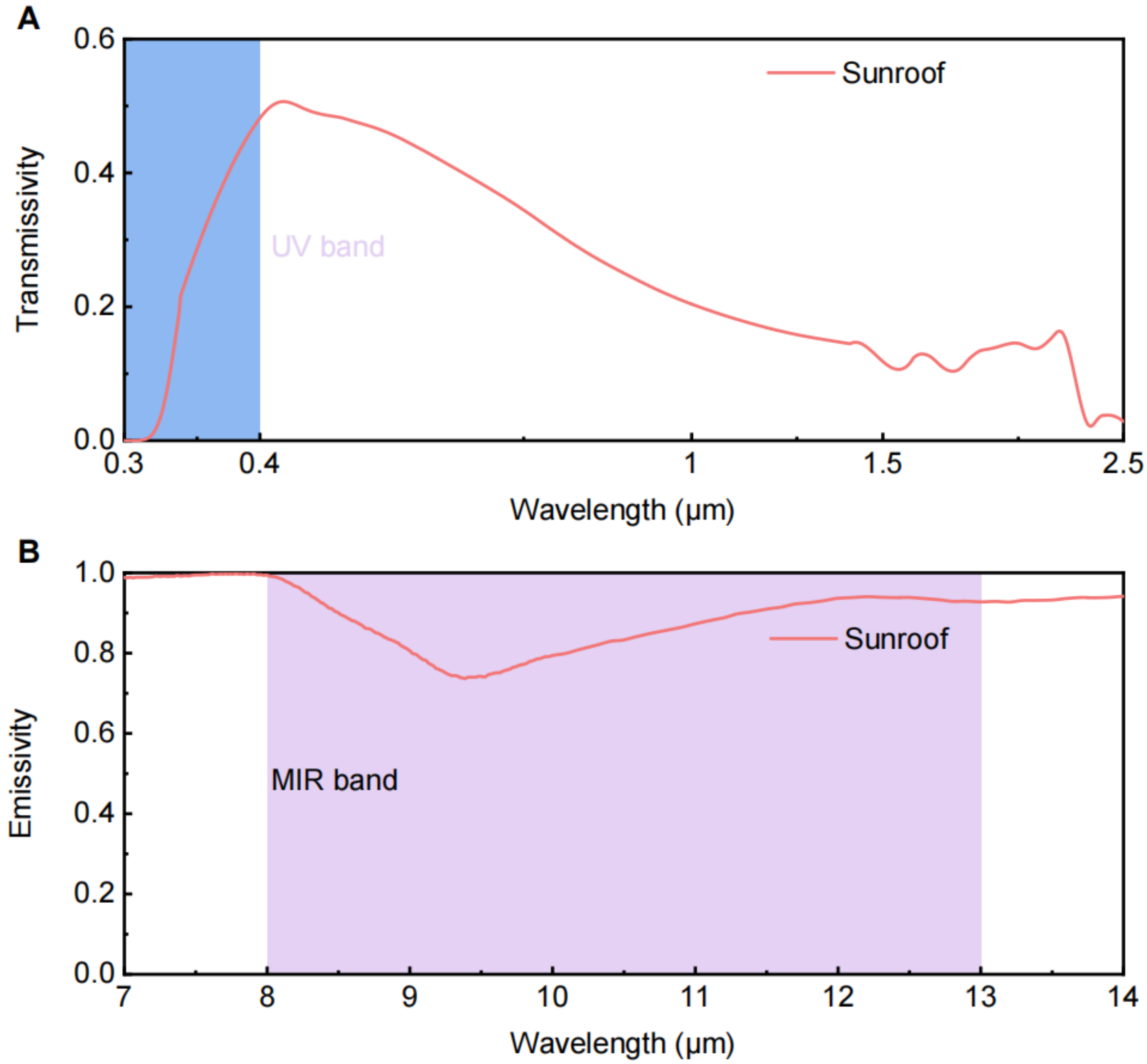

Supplementary Fig. 1. Spectral properties of the low-E sunroof. A, The transmissivity of sunroof in the solar band. **B,** The emissivity of sunroof in the MIR band.

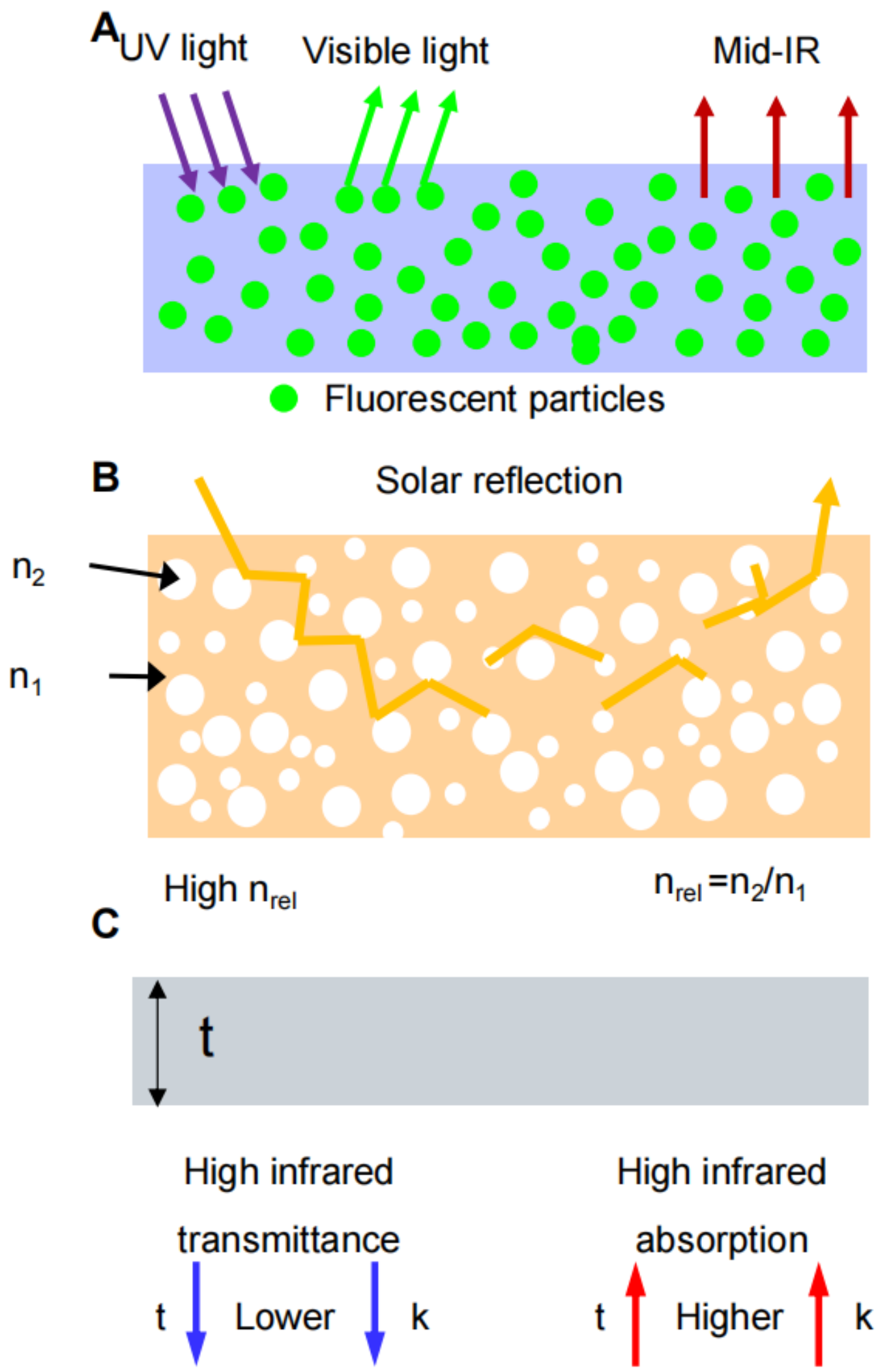

Supplementary Fig. 2. Hierarchical and photoluminescent structural design of metafabric sunshade. A, Polymer-based materials with high refractive index imaginary values have high loss capacity (absorption and emission capacity) for electromagnetic waves in the mid-infrared band. **B,** Micro-nano optical metamaterial structures with high refractive index contrast provide excellent reflection of wavelengths in the solar wavelength band. **C,** High transmission or emission function in the mid-infrared wavelength, with a gain effect on the emission of thermal radiation wavelengths.

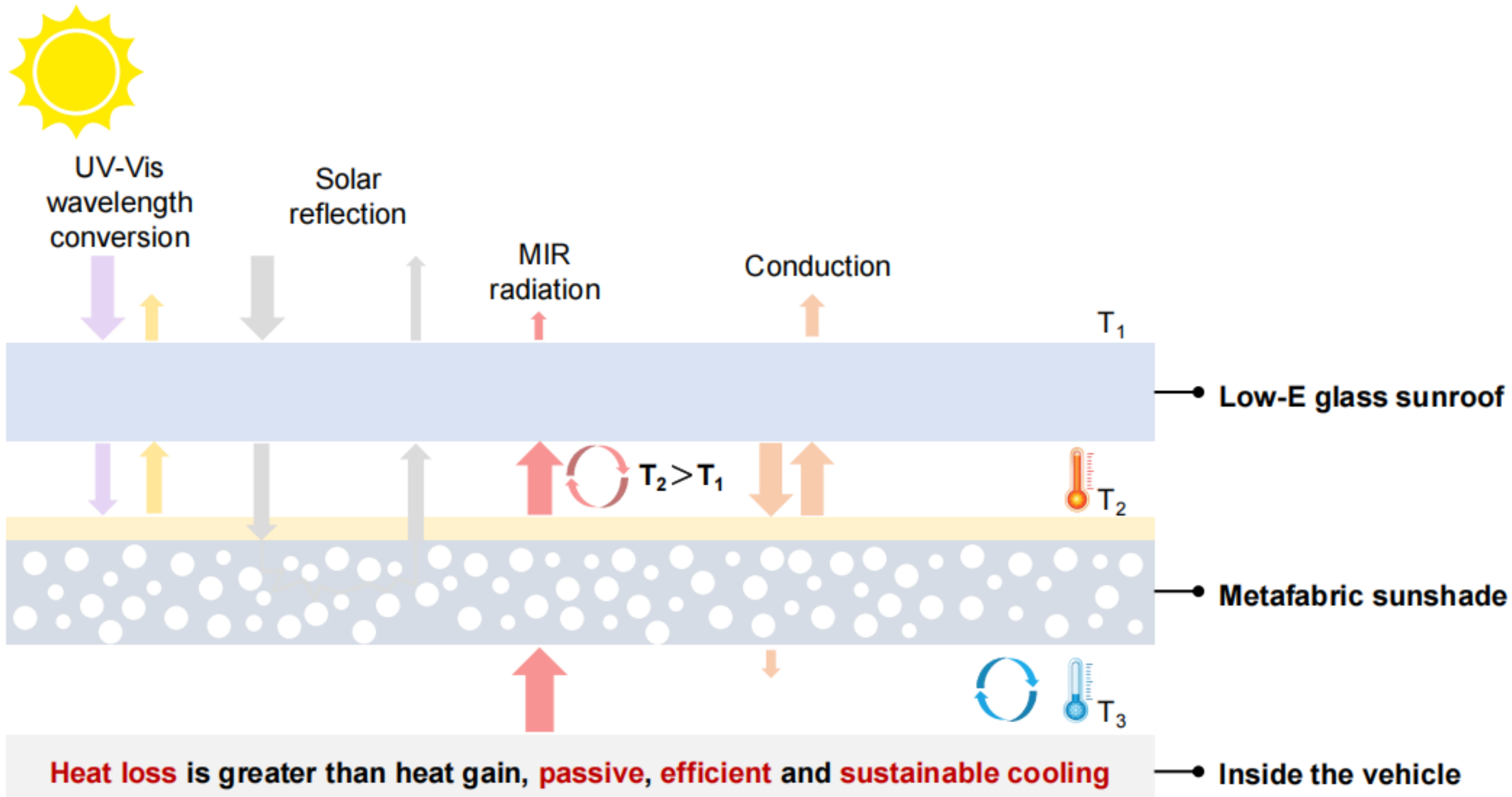

Supplementary Fig. 3. Passive and efficient cooling mechanism by using metafabric sunshade for the interior of vehicle sunroofs.

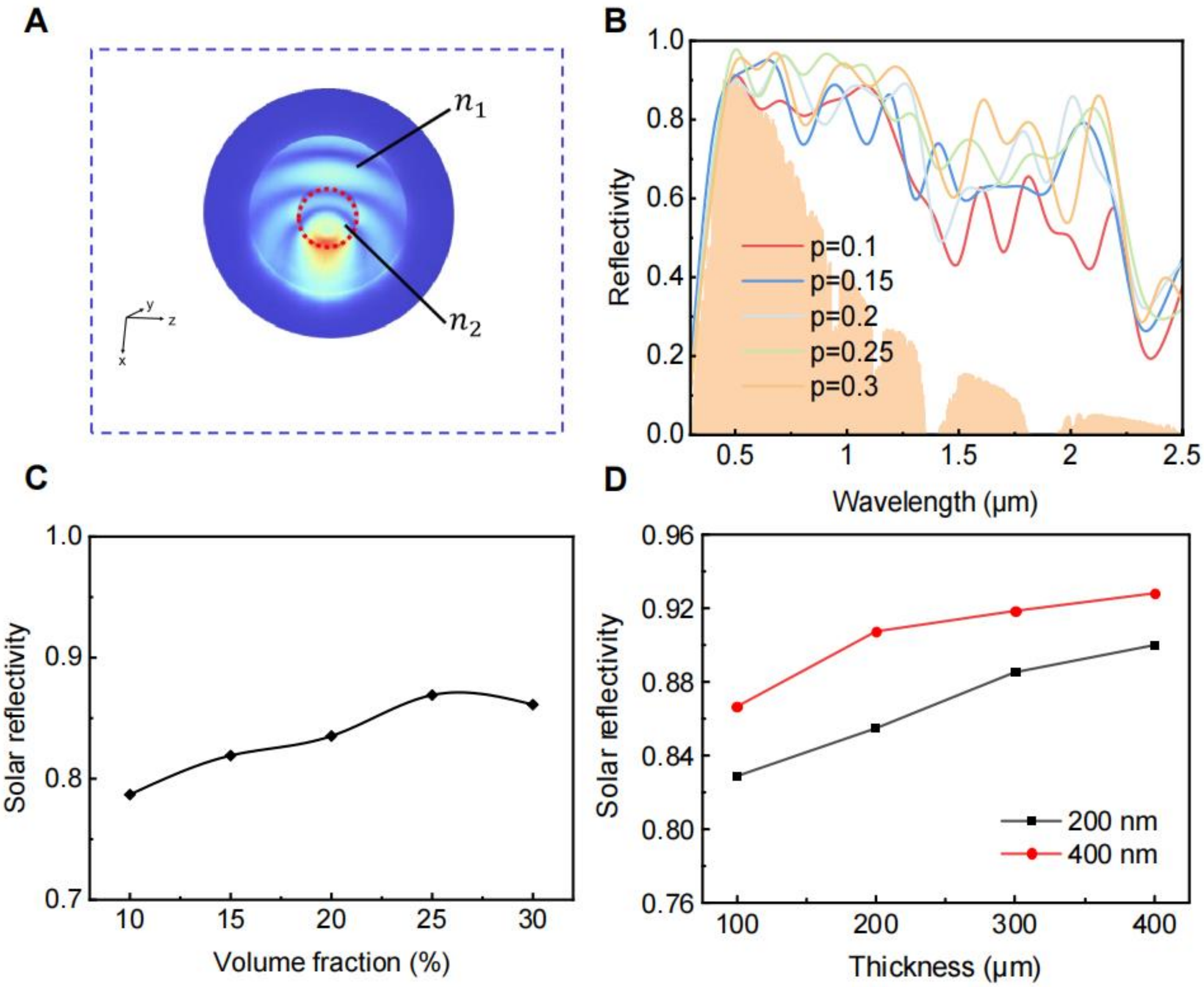

Supplementary Fig. 4. Reflectivity versus thickness and concentration for physical models of multi-media with $n_2 > n_1$. A, Light field analysis of individual micro/nano particles (n_2). The particle diameter is 400 nm. The incident wavelength is 600 nm. **B,** Variation of reflectivity with wavelength for TiO_2 doped concentrations of 0.1, 0.15, 0.2, 0.25, 0.3 (Normalized description). **C,** Variation of solar reflectivity with wavelength for TiO_2 doped concentrations of 10%, 15%, 20%, 25%, 30%. **D,** Solar reflectivity versus thickness for metafabric sunshade models with TiO_2 particle sizes of 200 nm and 400 nm.

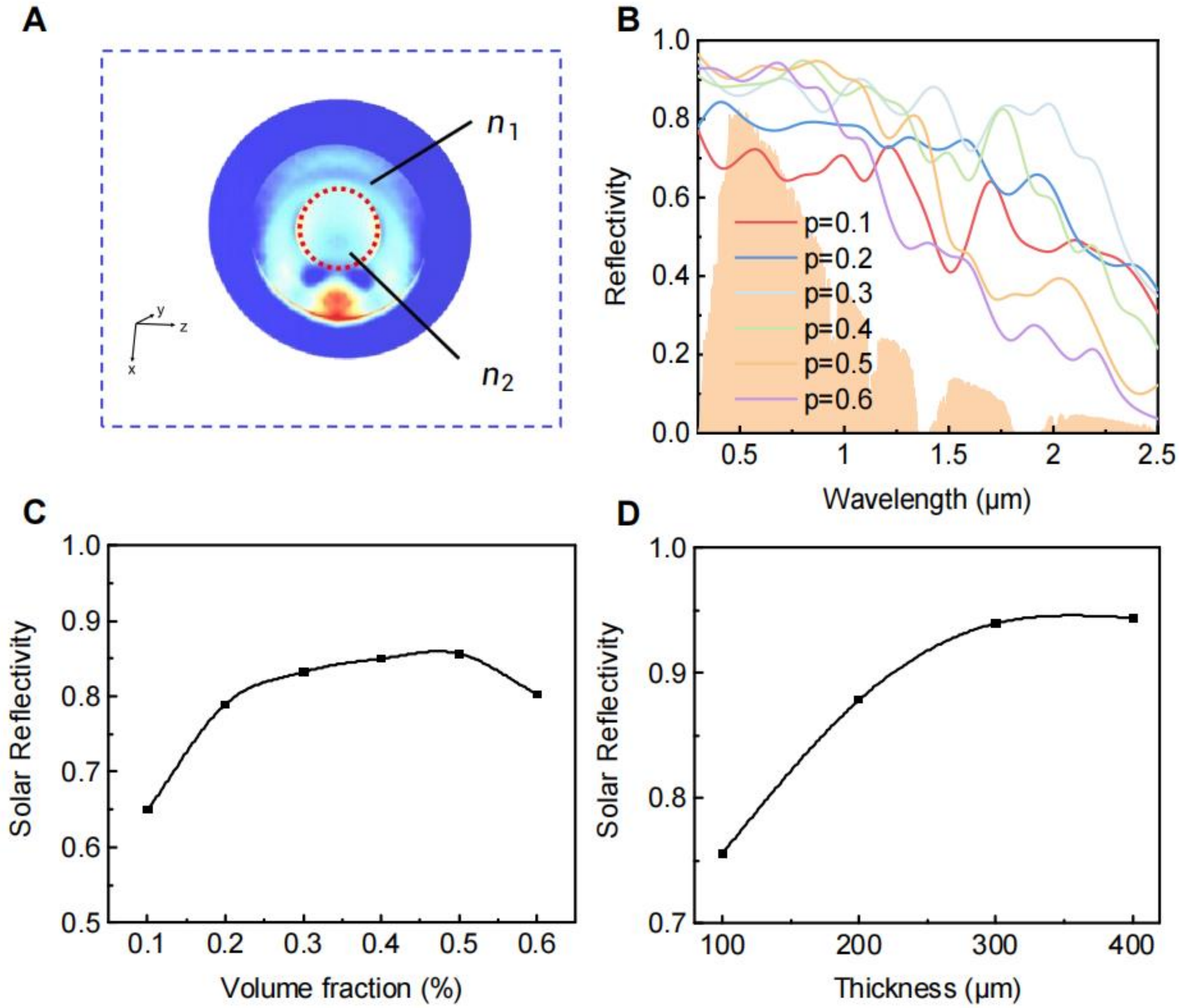

Supplementary Fig. 5. Reflectivity versus thickness and concentration for physical models of multi-media with $n_2 < n_1$. A, Light field analysis of individual micro/nano particles (n_3). The particle diameter is 400 nm. The incident wavelength is 600 nm. **B,** Variation of reflectivity with wavelength for micro/nano cavities doped concentrations of 0.1, 0.2, 0.3, 0.4, 0.5 (Normalized description). **C,** Variation of solar reflectivity with wavelength for micro/nano cavities doped concentrations of 10%, 20%, 30%, 40%, 50%. **D,** Solar reflectivity versus thickness for metafabric sunshade models with micro/nano cavities particle sizes of 400 nm.

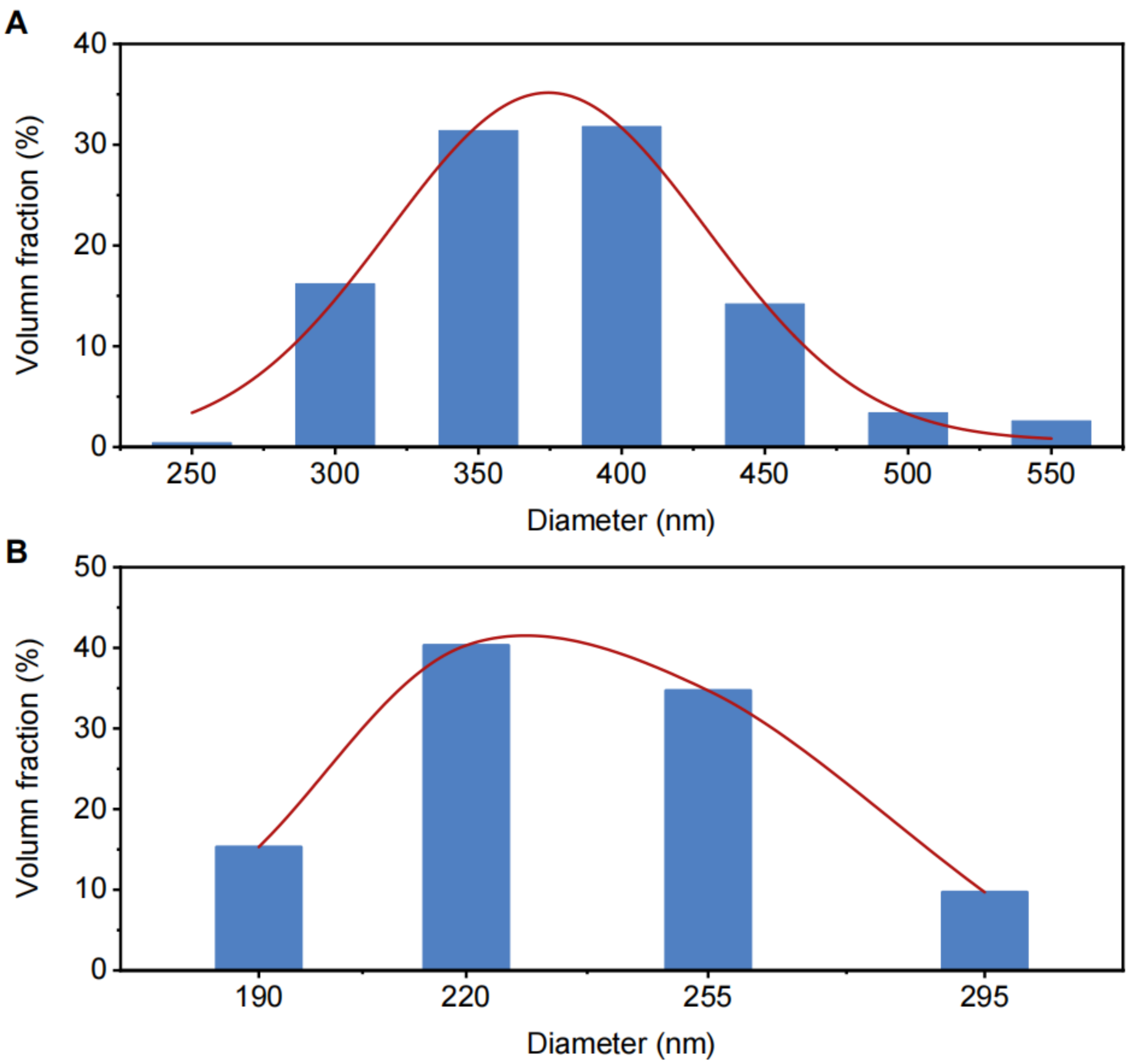

Supplementary Fig. 6. Size distribution of doped particles in metafabric sunshade. A, Particle size distribution of TiO_2 in the reflection layer of metafabric sunshade. **B,** Particle size distribution of fluorescent particles in the emission layer.

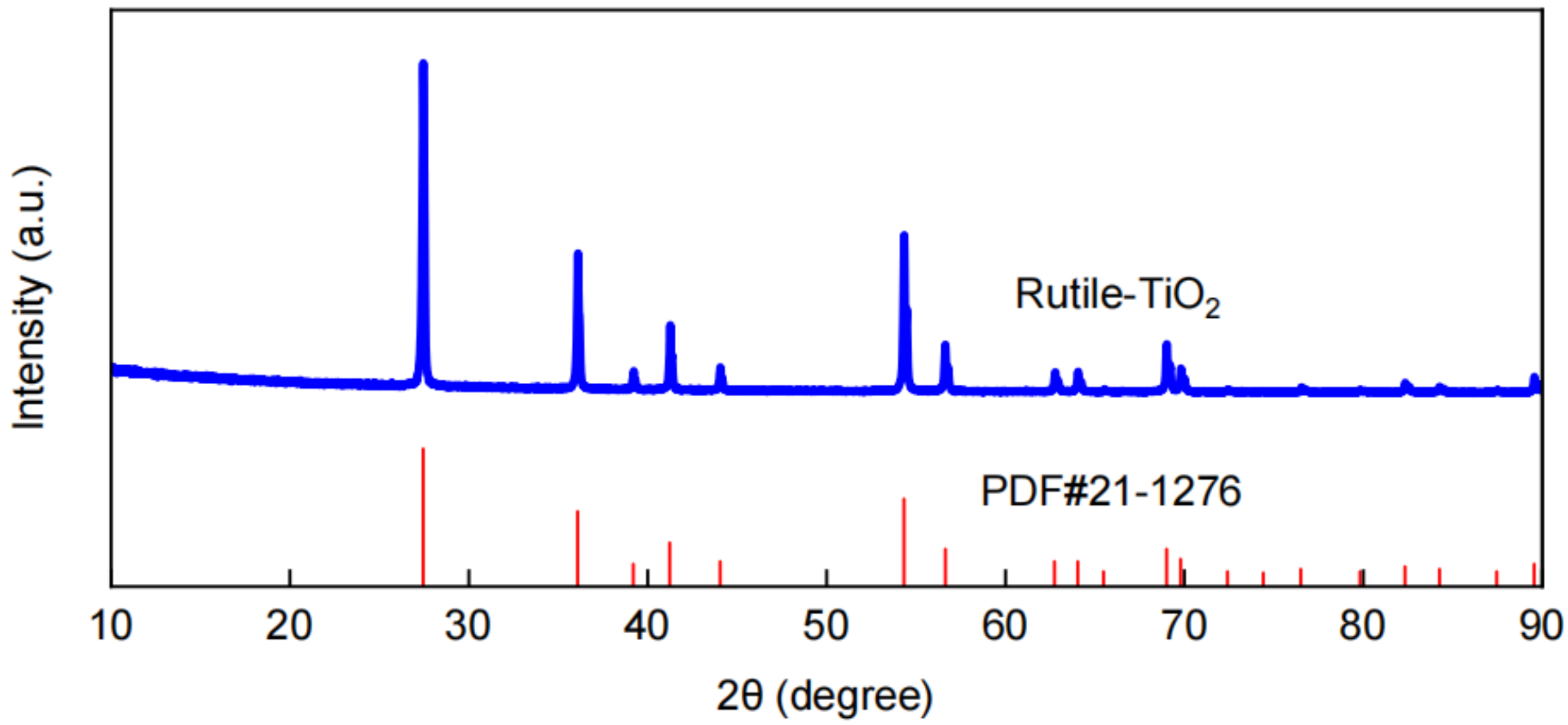

Supplementary Fig. 7. The lattice structure of rutile phase titanium dioxide employed in this study.

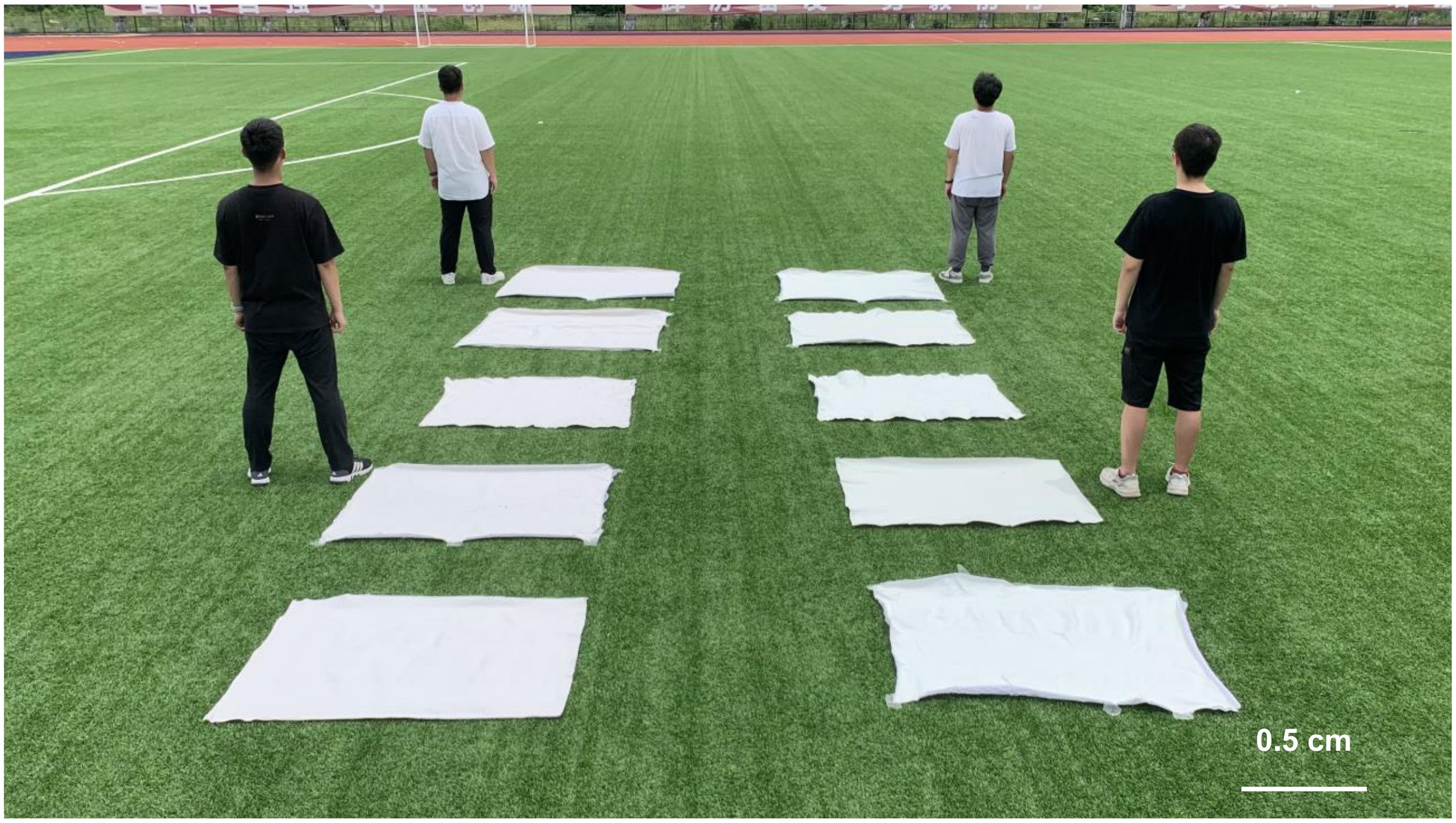

Supplementary Fig. 8. Large samples of metafabric sunshade and metafabric sunshade without FPs.

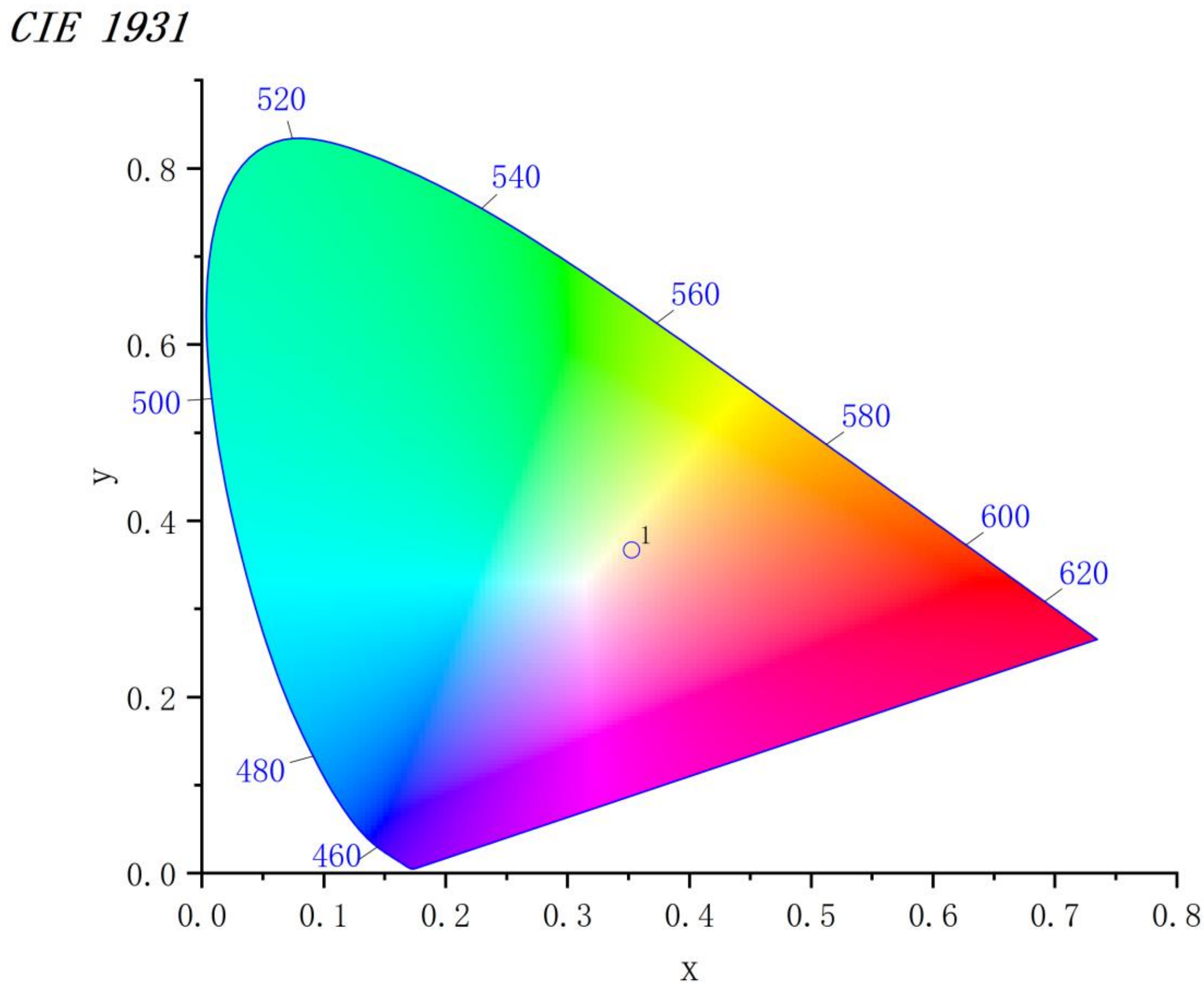

Supplementary Fig. 9. CIE color coordinates of metafabric sunshade.

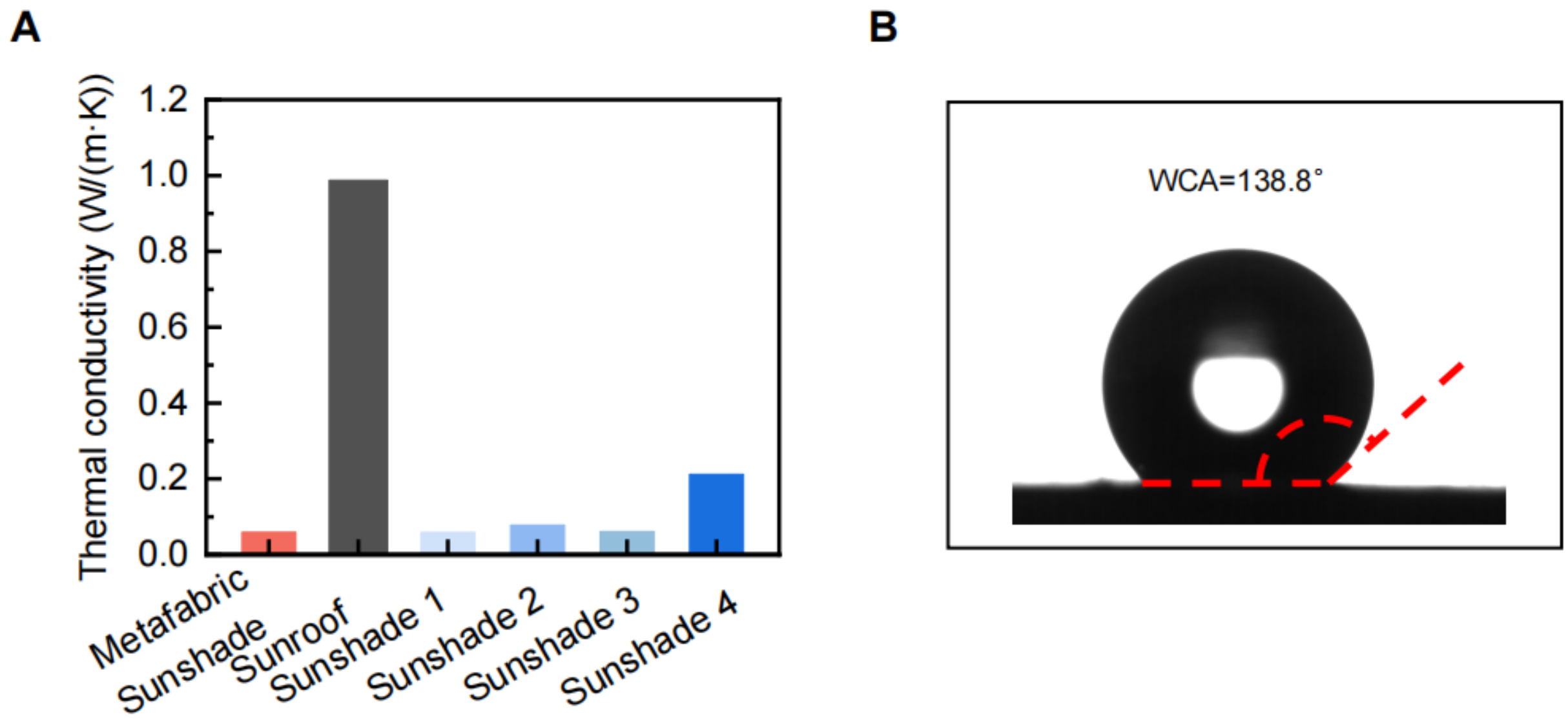

Supplementary Fig. 10. Thermal Conductivity and Hydrophobicity of Metafabric Sunshade. A, Thermal conductivity for sunroof, metafabric sunshade and sunshades. **B,** Metafabric sunshade's excellent hydrophobic angle.

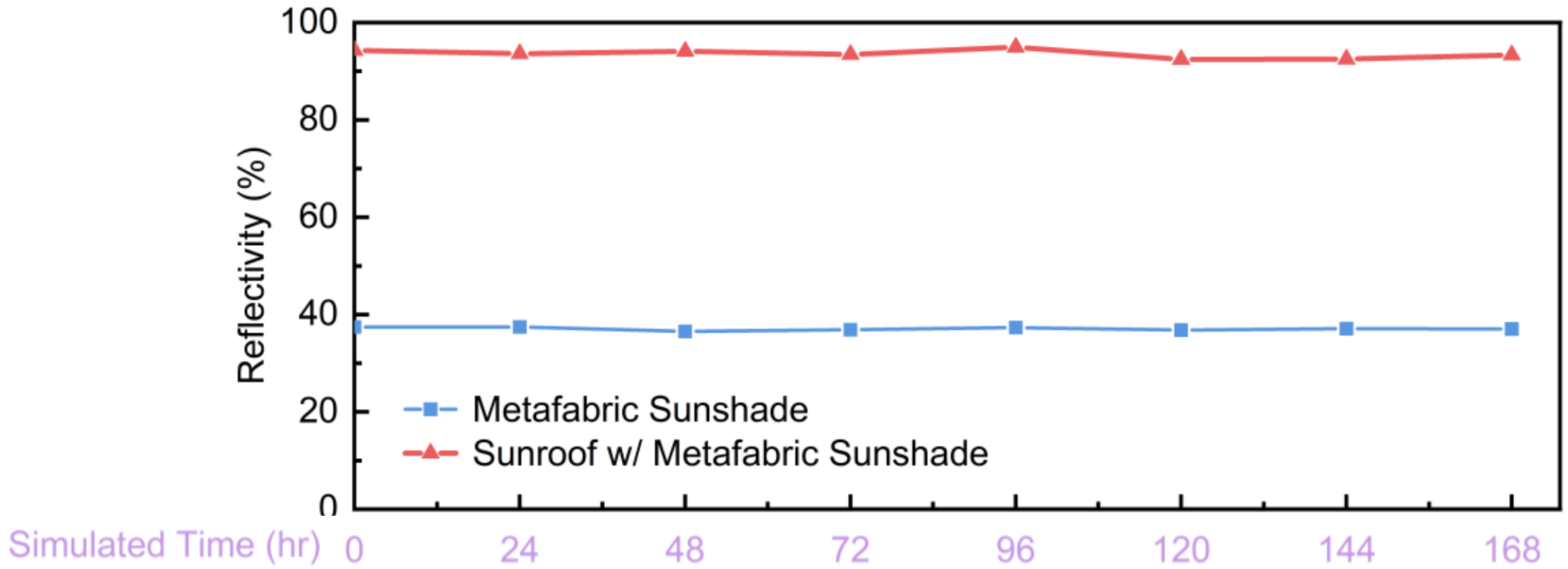

Supplementary Fig. 11. UV-radiation ageing test of the metafabric sunshade .

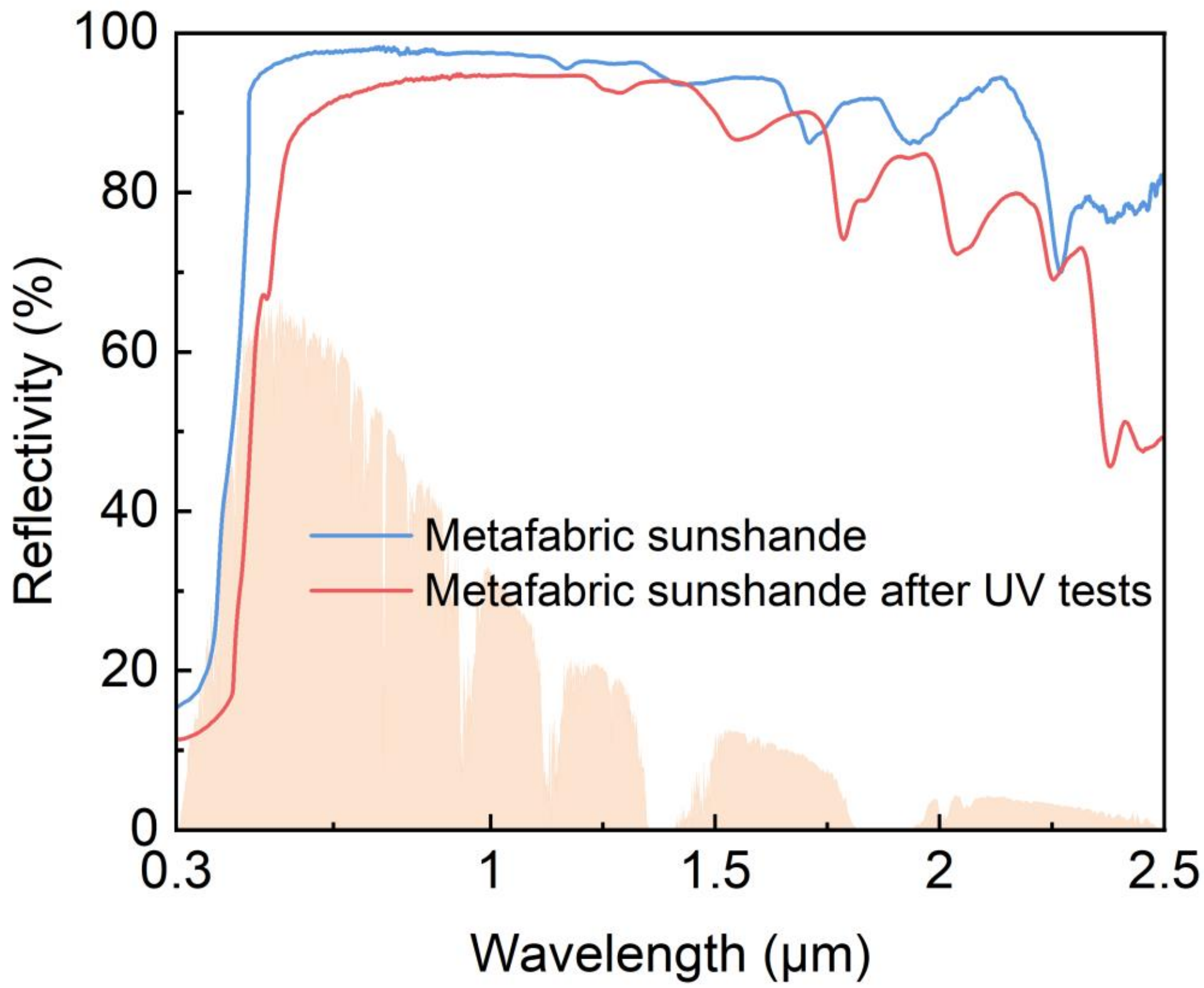

Supplementary Fig. 12. UV exposure and water spray cycle test with the GB/T 31899 standard.

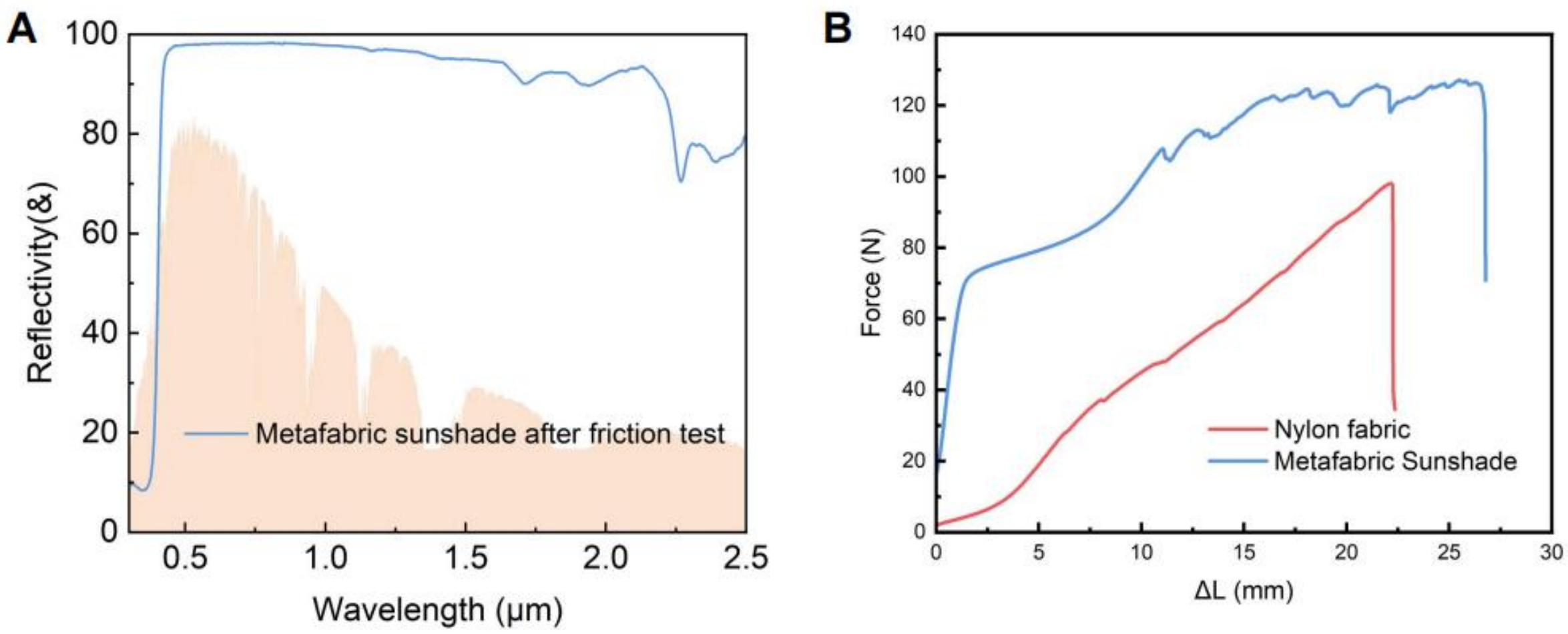

Supplementary Fig. 13. mechanical property test. **A**, solar reflectivity spectrum of metafabric sunshade after friction tests. **B**,tensile force–displacement curves of nylon fabric and metafabric sunshade.

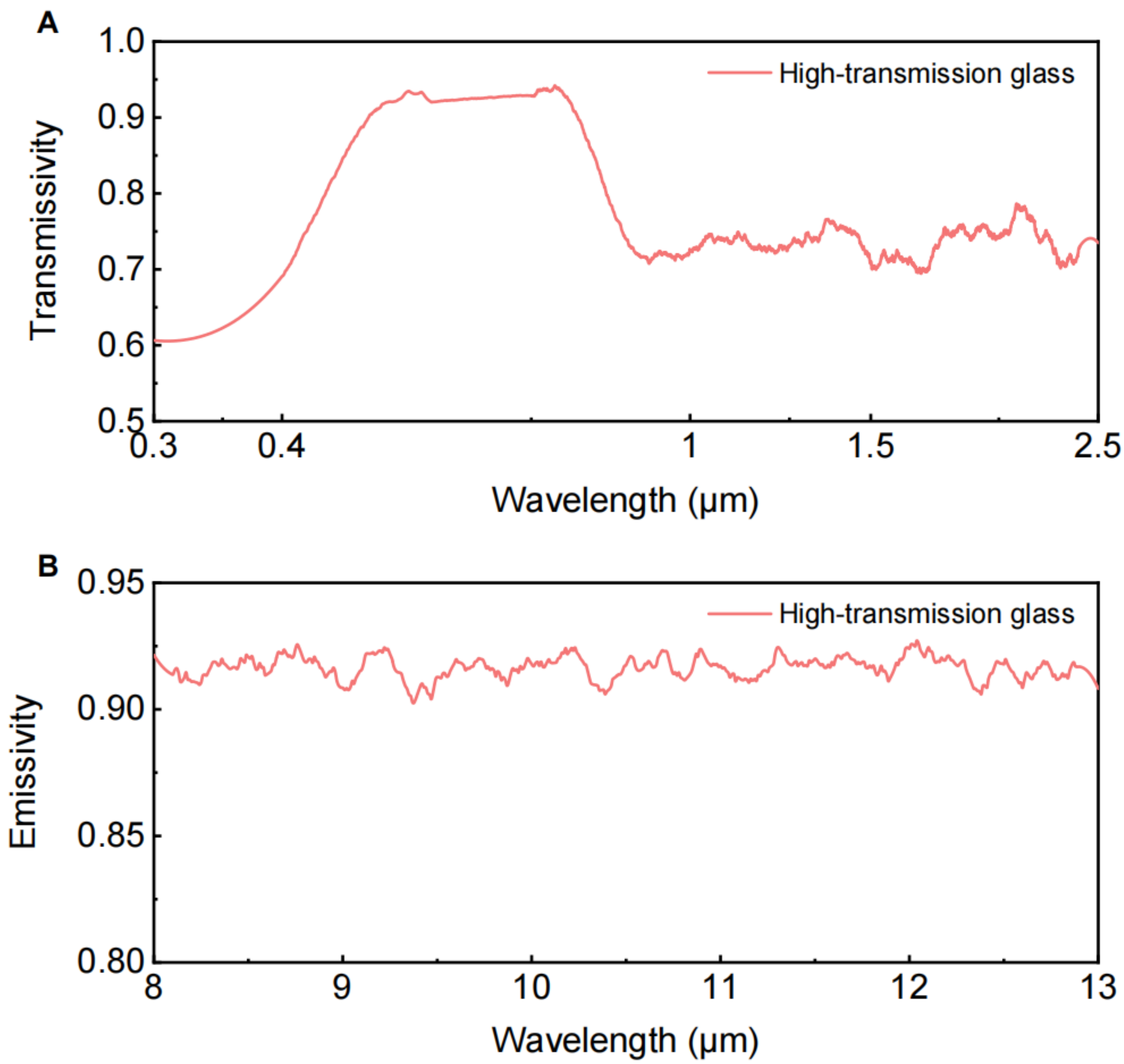

Supplementary Fig. 14. Spectral data of visible high-transmittance glass. A, Transmissivity data for visible high-transmittance glass. **B,** Emissivity data for visible high-transmittance glass.

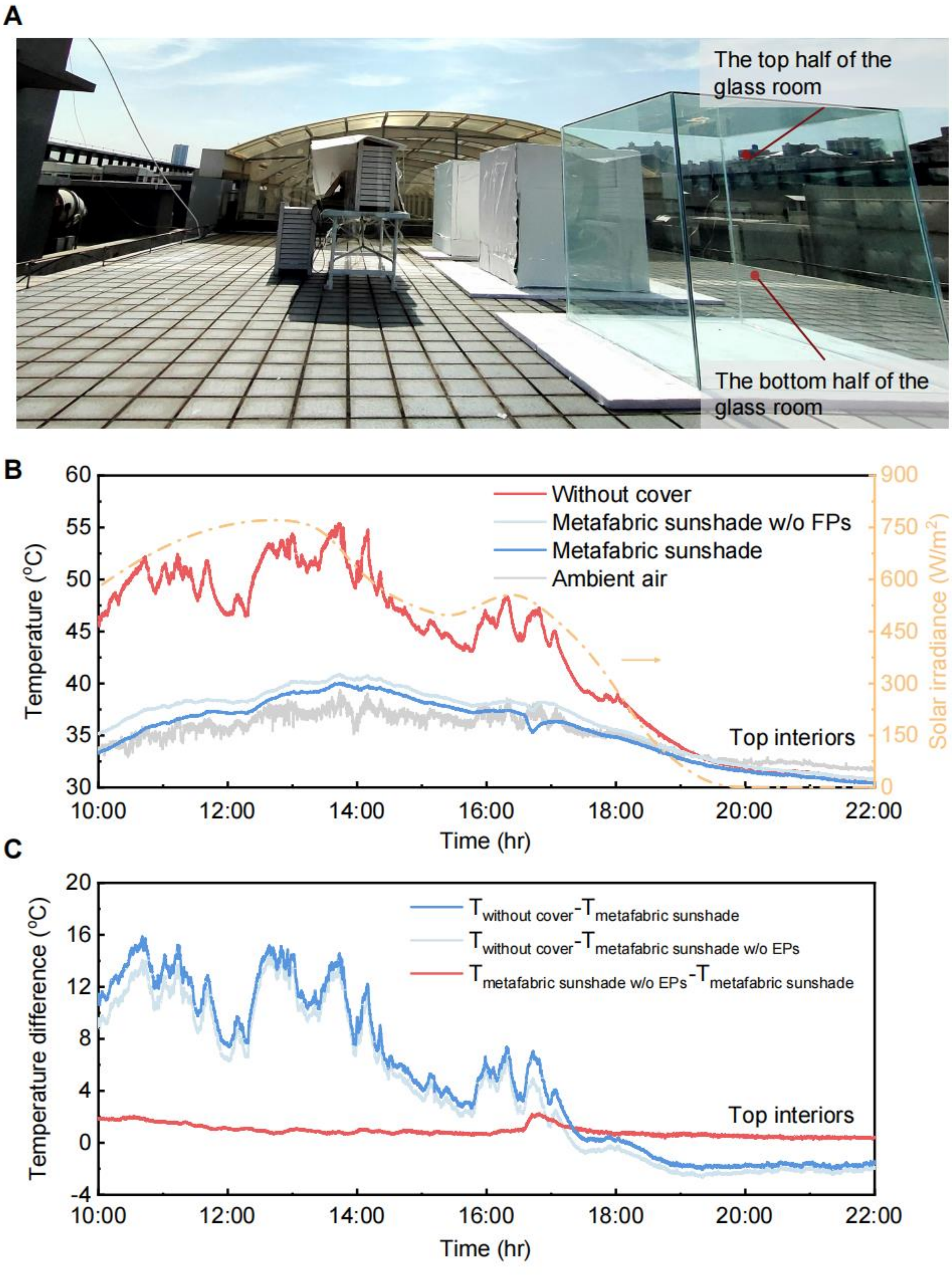

Supplementary Fig. 15. Cooling experiment applied to the top interiors of a glass room. A , Glass room cooling experiment diagram. **B,** A 12-hour continuous temperature measurement of

the subambient cooling performance test in Wuhan, China (114°41N′, 30°51′E, July 23-24, 2023). **C,** The temperature difference between the glass without cover, metafabric sunshade, and metafabric sunshade without FPs.

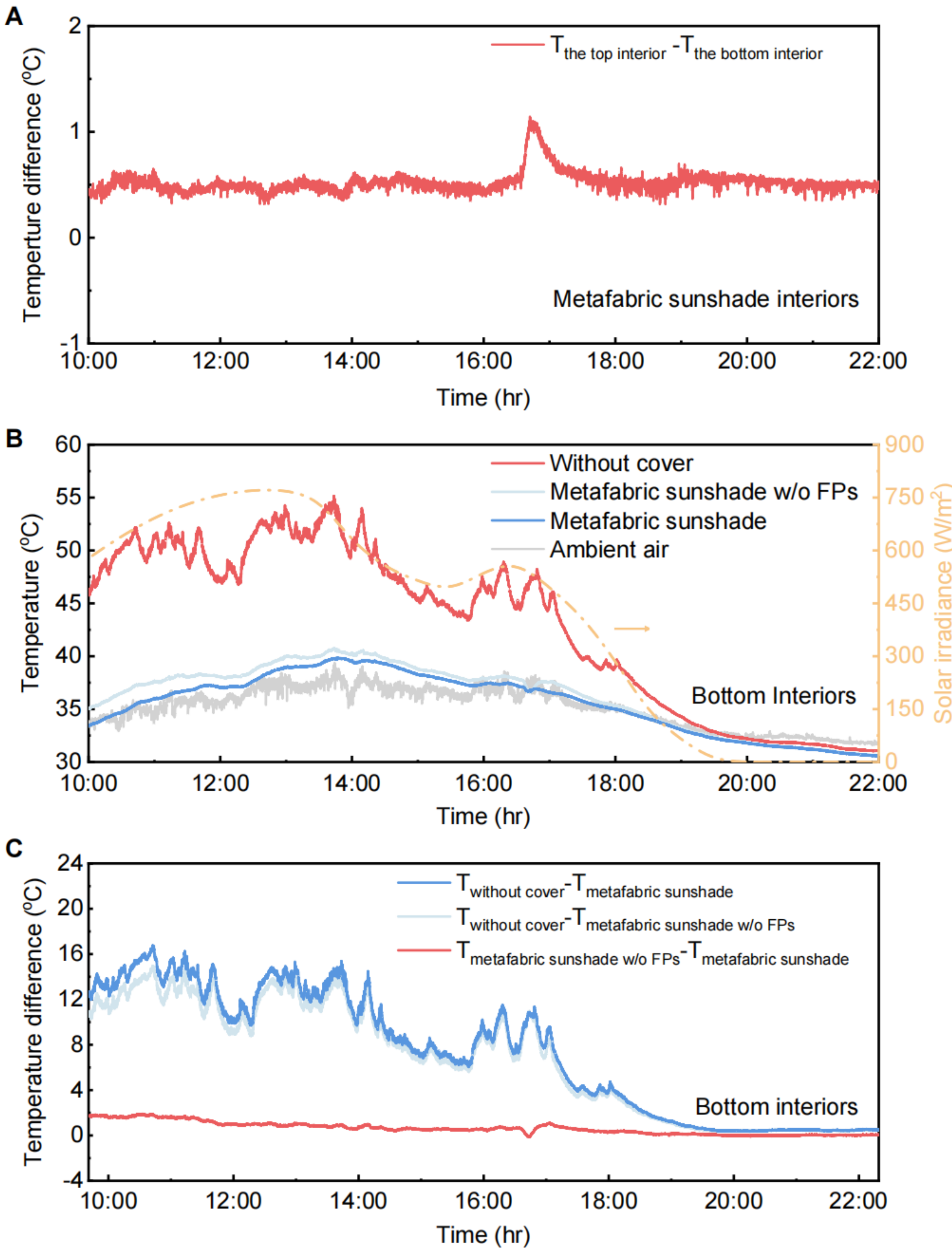

Supplementary Fig. 16. Cooling experiment applied to the top and bottom interiors of a glass room. A, The temperature difference between the top and bottom of a glass room with metafabric sunshade. **B,** A 12-hour continuous temperature measurement of the subambient cooling

performance test in Wuhan, China (114°41N′, 30°51′E, July 23-24, 2023). **C,** Temperature difference in the bottom interiors during indoor simulated cooling experiments under direct outdoor sunlight.

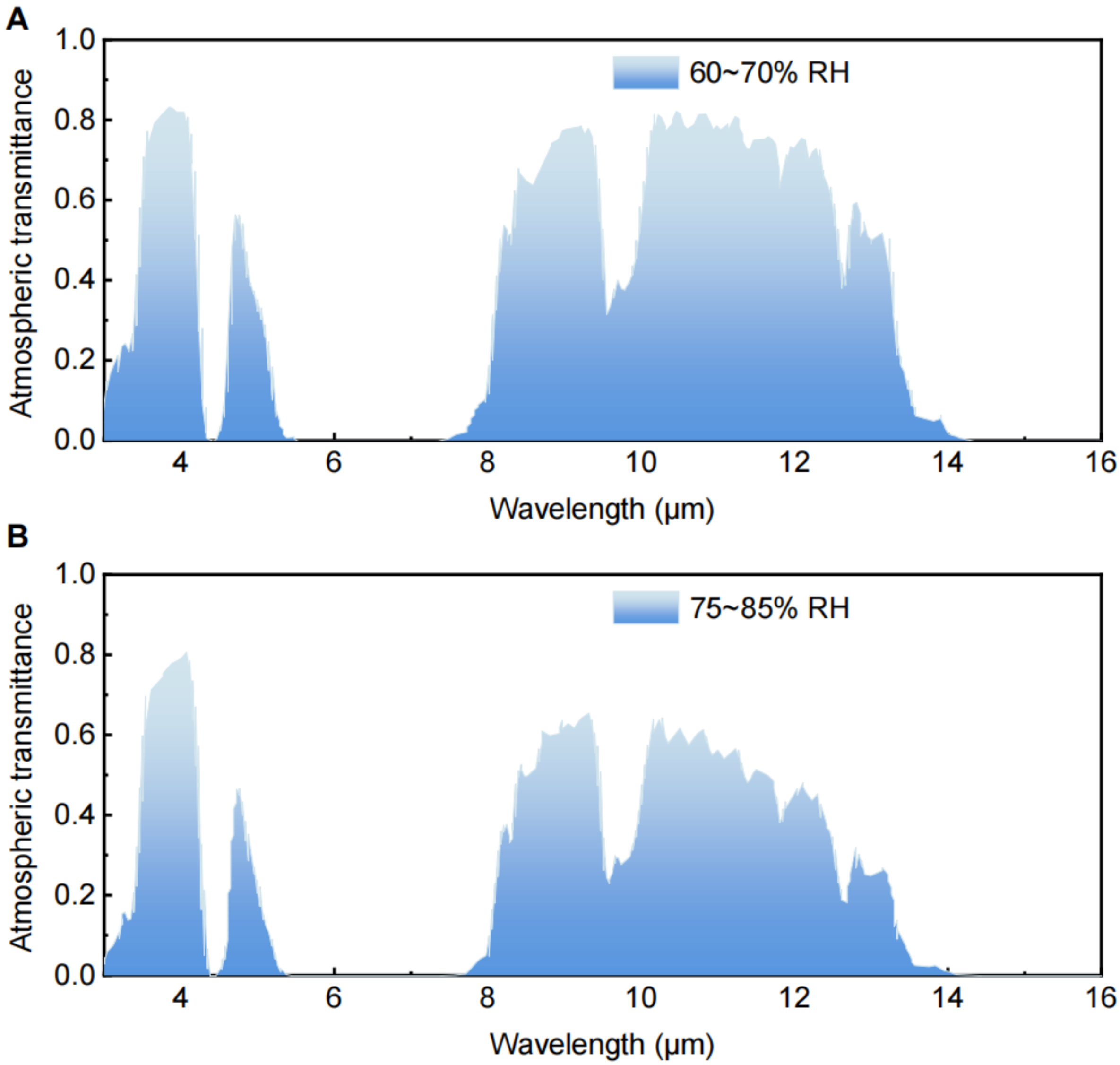

Supplementary **Fig. 17. Atmospheric transmittance for glass room and vehicle cooling experiments. A,** Atmospheric transmittance over Xishuangbanna Dai Autonomous Prefecture, Yunnan Province, China, February 23, 2024. **B,** Atmospheric transmittance over Wuhan, Hubei Province, China, August 3-6, 2024.

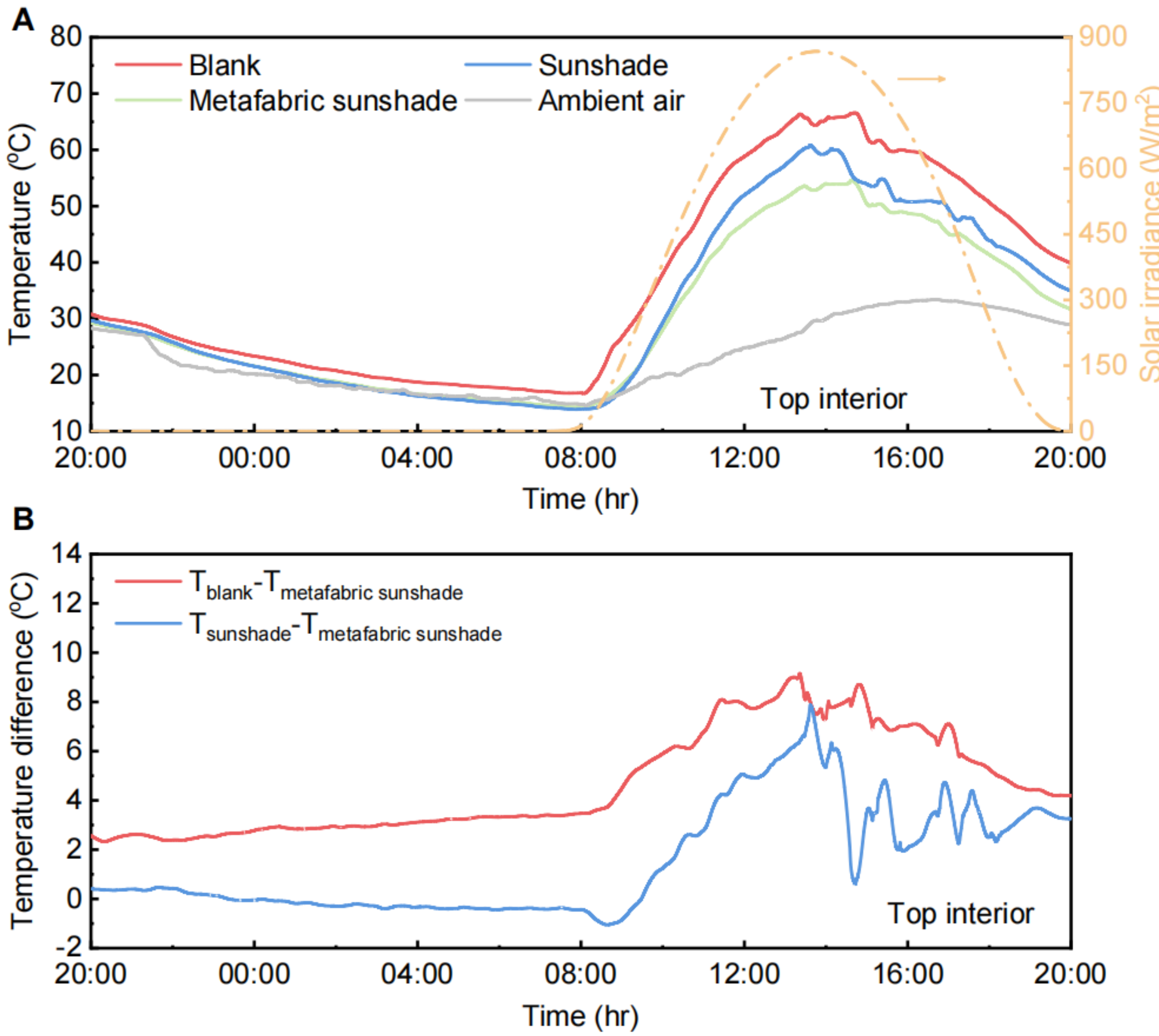

Supplementary Fig. 18. Temperature data plot of the upper side of the interior of vehicles with blank, commercial sunshade, and metafabric sunshade, respectively, located in Sipsongpanna, China (21°45' N, 100°36' E, February 22-23, 2024).

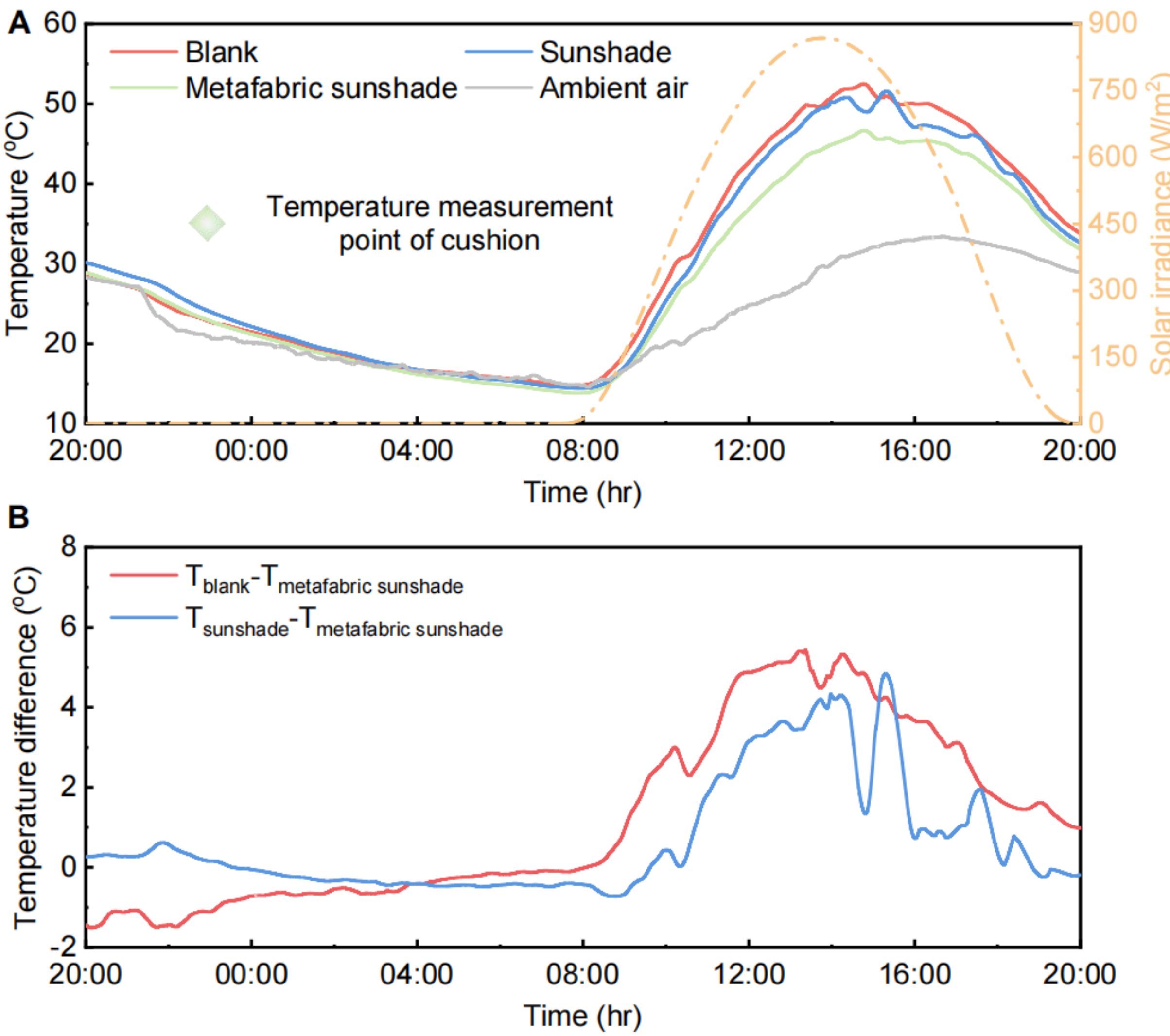

Supplementary Fig. 19. Temperature data plot of the cushion of vehicles with a blank, commercial sunshade, and metafabric sunshade, respectively, located in Sipsongpanna, China (21°45’ N, 100°36’ E, February 22-23, 2024).

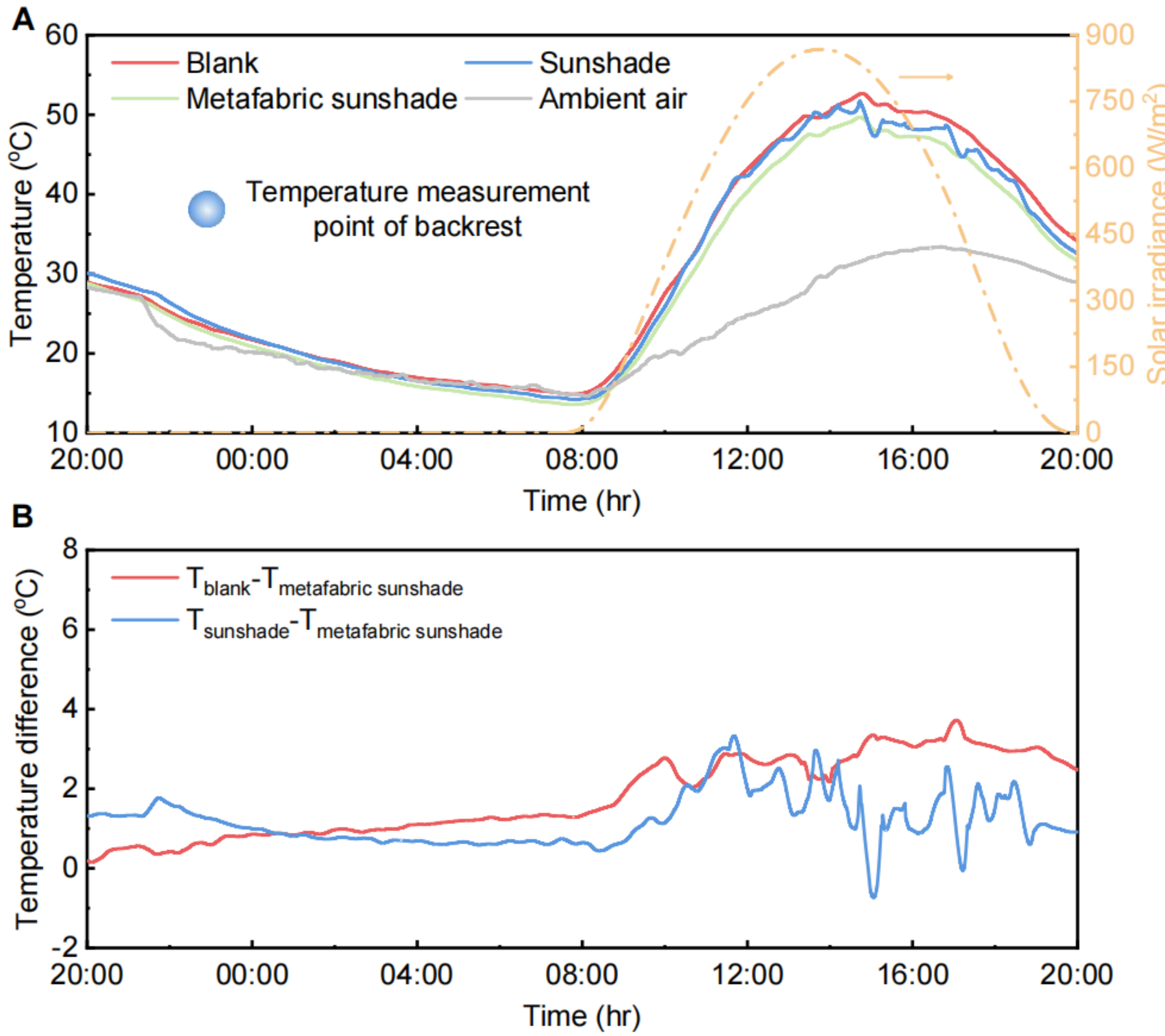

Supplementary Fig. 20. Temperature data plot of the backrest of vehicles with a blank, commercial sunshade, and metafabric sunshade, respectively, located in Sipsongpanna, China (21°45’ N, 100°36’ E, February 22-23, 2024).

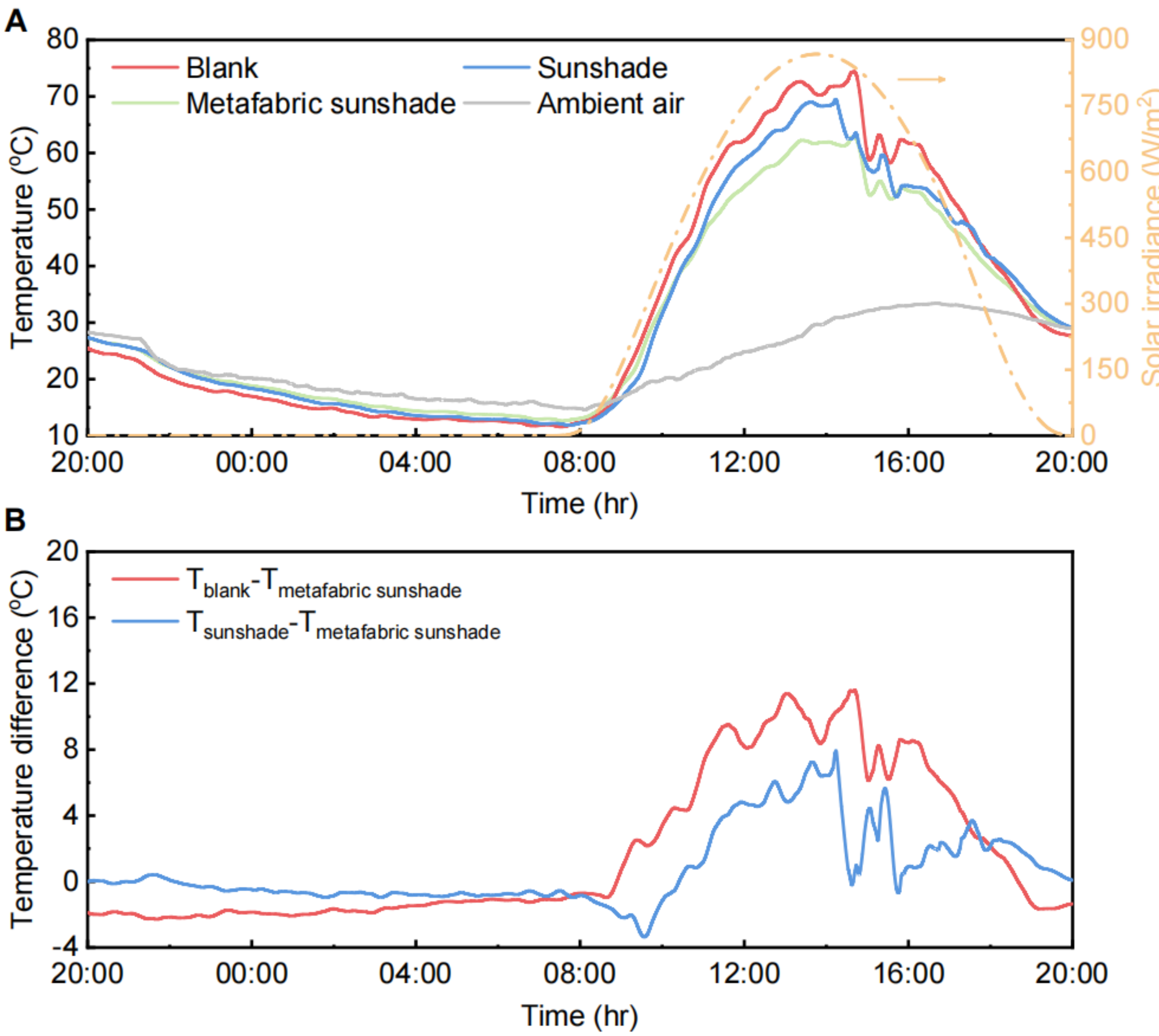

Supplementary Fig. 21. Temperature data plots of the sunroof and sunshade facing the interior side of vehicles with blank, commercial sunshade, and metafabric sunshade, respectively, located in Sipsongpanna, China (21°45’ N, 100°36’ E, February 22-23, 2024).

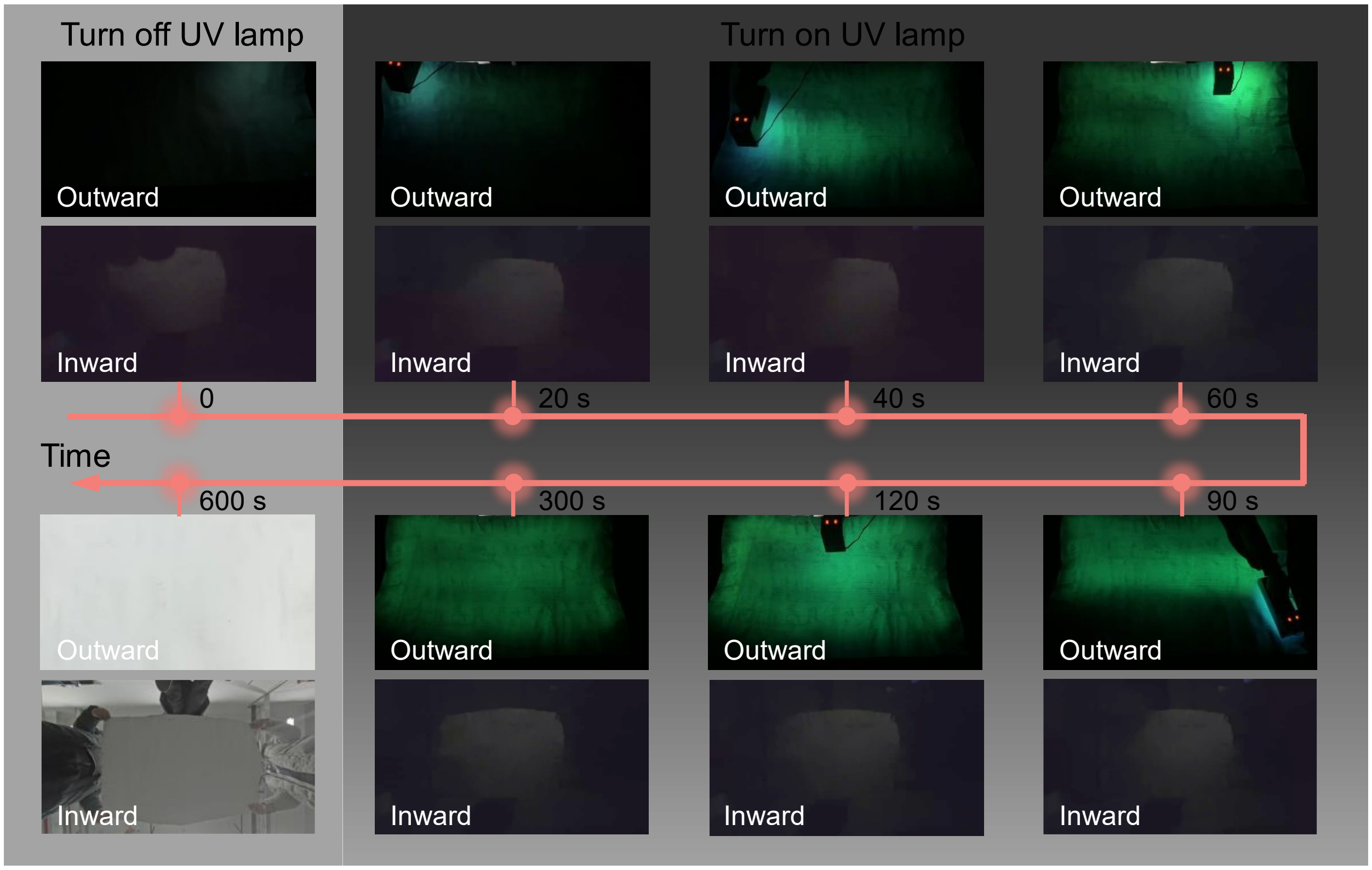

Supplementary Fig. 22. Demonstration image of photoluminescent metafabric sunshade outward surface under simulated UV lamp (365 nm) of solar radiation.

Supplementary Table 1. Spectroscopic data for sunroof, metafabric sunshade and commercial sunshades.

Simple	Materials and designs	Solar reflectivity (0.3-2.5 μm)	Average emissivity (8-13 μm)	Thickness
Sunroof	Glass	20.86	86.74	~5 cm
Metafabric sunshade	P(VdF-HFP) +TiO_2+Fluorescent particle	94.3	95.8	~ 340 μm
Metafabric sunshade without FPs	P(VdF-HFP) +TiO_2	93.1	94.9	~300 μm
Sunshade 1	Polyester	57.2	90.9	~ 789 μm
Sunshade 2	Polyester + Ag film	66.5	90.7	~ 922 μm
Sunshade 3	Al film + Transparent plastic particle	79.2	66.2	~ 1913 μm
Sunshade 4	Titanium silver gel+polyester+cotton Yarn	66.6	58.1	~ 455 μm

Supplementary Table 2. Solar reflectivity for composite sample with sunroof (outside), metafabric sunshade and commercial sunshades (inside).

Simple	Materials and designs	Solar reflectivity (0.3-2.5 μm)
Sunroof	Glass	20.86
Sunroof w/ metafabric sunshade	Glass+P(VdF-HFP) +TiO_2+Fluorescent particle	37.4
Sunroof with Sunshade 1	Glass+Polyester	29.3
Sunroof with Sunshade 2	Glass+Polyester + Ag film	22.8
Sunroof with Sunshade 3	Glass+Al film + Transparent plastic particle	32.6
Sunroof with Sunshade 4	Glass+Titanium silver gel+polyester+cotton Yarn	21.5

Supplementary Table 3. Costing of Metafabric sunshade.

Materials	Cost ($) per 1.5 m×1 m×340 μm
P(Vdf-HFP)	0.75
Fluorescent particles	2.02
TiO^2	2.19
DMF	2.39
Sodium dodecvl benzene sulfonate (SDBS)	0.25
Metafabric sunshade	12.27

Supplementary Table 4. Cost comparison between Metafabric sunshade and other commercial shades.

Sunshade	Materials	Cost ($)	Volume
Metafabric sunshade	P(VdF-HFP) +TiO2+Fluorescent particles	12.27	1.5 m×2 m×340 μm
Sunshade 1	Polyester	1.45	1.5 m×2 m×789 μm
Sunshade 2	Polyester + Ag film	75.01	1.5 m×2 m×922 μm
Sunshade 3	Al film + Transparent plastic particle	23.41	1.5 m×2 m×1913 μm
Sunshade 4	Titanium silver gel+polyester+cotton Yarn	16.52	1.5 m×2 m×455 μm

Supplementary Table 5. Actual glass room cooling test net cooling power calculation parameters (Atmospheric transmittance over Wuhan, Hubei Province, China, August 3-6, 2024).

Thermal conductivity of air (λ_{air})	0.026 (W/(m·K))
Kinematic viscosity of air (v_{air})	1.6×10^{-5} (m^2/s)
Prandtl number of air(P_r)	0.7
Coefficient of thermal expansion of air (β, Tamb=310.55 K)	1/310.55
Wind speed of air (U)	1.6~3.3 m/s
Relative humidity	81%
Average environment temperature (T_{amb})	310.55 K
Average sample temperature ($T_{metafabric}$)	314.95 K
Average temperature difference (T_{amb}-$T_{metafabric}$)	-4.4 K
Characteristic length (L)	1 m
Acceleration of gravity (g)	9.8 m/s

Supplementary Table 6. Actual vehicle cooling test net cooling power calculation parameters (Atmospheric transmittance over Xishuangbanna Dai Autonomous Prefecture, Yunnan Province, China, February 23, 2024).

Thermal conductivity of air (λ_{air})	0.026 (W/(m·K))
Kinematic viscosity of air (v_{air})	1.6×10^{-5} (m^2/s)
Prandtl number of air(P_r)	0.7
Coefficient of thermal expansion of air (β, Tamb=302.15K)	1/302.15
Wind speed of air (U)	1.5~3 m/s
Relative humidity	70%
Average environment temperature (T_{amb})	302.15 K
Average sample temperature ($T_{metafabric}$)	320.52 K
Average temperature difference (T_{amb}-$T_{metafabric}$)	-18.37 K
Characteristic length (L)	1 m
Acceleration of gravity (g)	9.8 m/s

Supplementary Video 1

Overview movie. Movie display of Photoluminescent-assisted Scalable Passive Cooling Hierarchical Metafabric Sunshade for sunburnt glass rooms and vehicles in extreme heat temperature. The movie demonstrates the scientific analysis of internal temperatures in glass rooms and vehicles equipped with low-E large sunroofs under extreme heat conditions. Furthermore, it highlights the research significance and innovation of our study aimed at achieving efficient passive cooling of the interior through hierarchical and photoluminescent metafabric sunshade. Cooling tests demonstrated that the metafabric sunshade in the vehicle interior resulted in a significant temperature reduction of 17.8℃ and 14.4℃ compared to the vehicles with blank and commercial sunshade, respectively. Moreover, this breakthrough in metafabric sunshade technology exhibits significant potential for enhancing passive thermal management applications such as glass rooms, vehicles, and outdoor shelters.

Supplementary Video 2

The scalable scraping process of metafabric sunshade.

Supplementary Video 3

Demonstration of the photoluminescent and non-pure white capability in metafabric sunshade.

Supplementary Video 4

Demonstration of the photoluminescent capability in metafabric sunshade.

References

1. Chen, M. *et al*., Designing mesoporous photonic structures for high-performance passive daytime radiative cooling. *Nano Lett.* **21**, 1412-1418 (2021).
2. Min, X. *et al.,* A smart thermal-gated bilayer membrane for temperature-adaptive radiative cooling and solar heating. *Sci. Bull.* **67**, 1874-1881 (2022).
3. Wang, T. *et al.*, A structural polymer for highly efficient all-day passive radiative cooling. *Nat.Commun.* **12**, 365 (2021).
4. Liu, C. *et al.*, Effect of atmospheric water vapor on radiative cooling performance of different surfaces. *Sol. Energy* **183**, 218-225 (2019).
5. Huang, J. *et al.*, Core-shell particles for devising high-performance full-day radiative cooling paint. *Applied Mater. Today* **25**, 101209 (2021).
6. Awbi, H. B. Calculation of convective heat transfer coefficients of room surfaces for natural convection. *Energy Buildings* **28**, 219-227 (1998).
7. Khalifa, A.-J. N. Natural convective heat transfer coefficient–a review: II. Surfaces in two- and three-dimensional enclosures. *Energy Convers. Manage.* **42**, 505-517 (2001).
